# Mature Biofilm Degradation by Potential Probiotics: *Aggregatibacter actinomycetemcomitans* versus *Lactobacillus* spp.

**DOI:** 10.1371/journal.pone.0159466

**Published:** 2016-07-20

**Authors:** Norzawani Jaffar, Yuya Ishikawa, Kouhei Mizuno, Toshinori Okinaga, Toshinari Maeda

**Affiliations:** 1 Department of Biological Functions Engineering, Graduate School of Life Science and Systems Engineering, Kyushu Institute of Technology, Wakamatsu-ku, Kitakyushu, Japan; 2 Faculty of Health Sciences, Gong Badak Campus, Universiti Sultan Zainal Abidin (UniSZA), Kuala Terengganu, Terengganu Darul Iman, Malaysia; 3 Department of Materials Science and Chemical Engineering, Kitakyushu National College of Technology, Kitakyushu, Japan; 4 School of Oral Health Sciences, Faculty of Dentistry, Kyushu Dental University, Kitakyushu, Japan; 5 Research Center for Advanced Eco-fitting Technology, Kyushu Institute of Technology, Kitakyushu, Japan; Agricultural University of Athens, GREECE

## Abstract

The biofilm degradation of *Aggregatibacter actinomycetemcomitans* is essential as a complete periodontal disease therapy, and here we show the effects of potential probiotic bacteria such as *Lactobacillus* spp. for the biofilm of several serotypes of *A*. *actinomycetemcomitans* strains. Eight of the 13 species showed the competent biofilm degradation of ≥ 90% reduction in biofilm values in *A*. *actinomycetemcomitans* Y4 (serotype b) as well as four of the seven species for the biofilm of *A*. *actinomycetemcomitans* OMZ 534 (serotype e). In contrast, the probiotic bacteria did not have a big impact for the degradation of *A*. *actinomycetemcomitans* SUNY 75 (serotype a) biofilm. The dispersed *A*. *actinomycetemcomitans* Y4 cells through the biofilm detachment were still viable and plausible factors for the biofilm degradation were not due to the lactic acid and low pH conditions. The three enzymes, protease, lipase, and amylase may be responsible for the biofilm degradation; in particular, lipase was the most effective enzyme for the biofilm degradation of *A*. *actinomycetemcomitans* Y4 along with the protease activity which should be also important for the other serotypes. Remarkable lipase enzyme activities were detected from some of the potential probiotics and a supporting result using a lipase inhibitor presented corroborating evidence that lipase activity is one of the contributing factors for biofilm degradation outside of the protease which is also another possible factor for the biofilm of the other serotype of *A*. *actinomycetemcomitans* strains. On the other hand, the biofilm of *A*. *actinomycetemcomitans* SUNY 75 (serotype a) was not powerfully degraded by the lipase enzyme because the lipase inhibitor was slightly functional for only two of potential probiotics.

## Introduction

*Aggregatibacter actinomycetemcomitans* is a gram-negative, non-motile, pathogenic oral bacterium that contributes to periodontal disease [1]. It is localized in the dental plaque, gingival crevices, and the buccal mucosa of up to 36% of the normal population [2, 3]. *A*. *actinomycetemcomitans* is one of the causative agents of periodontal disease such as juvenile localized periodontitis and the early onset of periodontitis [1] and might sometimes be accompanied by alveolar bone loss associated with bone defects and probing attachment loss [4]. In fact, the pathogen can express several virulence factors to survive in the oral cavity; these include leukotoxin, cytolethal distending toxin (Cdt) [5, 6], lipopolysaccharide (LPS), bone resorption-inducing toxins [7], and epitheliotoxin, which are known to be involved in the interaction between host cells [8]. Furthermore, *A*. *actinomycetemcomitans* and other members of the HACEK group of bacteria (*Haemophilus influenzae*, *H*. *parainfluenzae*, *Aggregatibacter aphrophilus*, *Cardiobacterium hominis*, *Eikenella corrodens*, and *Kingella kingae*) are primarily associated with infective endocarditis [9, 10]. It was proposed in a review article that periodontitis influences the host`s susceptibility to cardiovascular disease and preterm labor in three ways: 1) by shared risk factors, 2) via the periodontium acting as a reservoir for inflammatory mediators, and 3) by the subgingival biofilm acting as a reservoir for gram-negative bacteria (10). *A*. *actinomycetemcomitans* biofilm has been reported to form tenacious attachments on surfaces due to an existence of fimbriae [1], which are mediated by the tight-adherence (*tad*) gene loci *flp*, *rcpA*, and *rcpB* [11]. However, the true mechanism of this biofilm formation is not yet fully understood. For instance, a colony variant with very few fimbriae is still able to form a robust biofilm and adhere to surfaces [12]. It has become increasingly clear that bacterial activities in the oral cavity might ease the propagation of this pathogenic organism to other body parts, particularly in immunocompromised patients, such as those with diabetes, rheumatoid arthritis, or those receiving immunosuppressive treatment [13]. Antibiotics or nonsurgical therapies such as scaling and root planning are used to manage periodontal disease [14]. Unfortunately, systemic antibiotic usage definitely suppresses the periodontal microflora and has a limited effect against the targeted bacteria.

Furthermore, *A*. *actinomycetemcomitans* biofilm exhibits a higher resistance against antibiotic applications than planktonic cells [15]. Established biofilms can even be tolerant to antimicrobial agents at concentrations 10–1000-times higher than those needed to completely kill planktonic bacteria [16]; they are almost impossible to be phagocytized by immune cells due to the restricted penetration of immunity factors by extracellular polysaccharides [17], and they are sometimes not recognized by the hosts cells [18]. Phagocytes that attempt an assault on the biofilm might cause more harm to the surrounding tissues than to the biofilm itself [19]. By targeting biofilm degradation, an optimum effect may be achieved by the use of antimicrobials and the cell defense of the host. A bacteriotherapy approach using probiotic cells to counteract this activity has been extensively studied [20–23]. A probiotic, as defined by the World Health Organization (WHO, 2001), is a “live microorganism which, when administered in adequate amounts, confers a health benefit on the host” [24]. We chose probiotic bacteria for this study due to their wide spectrum of different effects including direct antagonism against a pathogen, improving gut health and enhancing the immunity response in humans [20]. Another salient property of probiotics is their ability to aggregate with another organism. For example, a pathogen, which might provide great advantages over non-aggregating microorganisms, can be easily removed from the intestinal environment [25]. These properties of probiotic bacteria make them a smart choice to promote the natural killing of a pathogen via bacterial interactions without affecting the normal flora at the site of infection. Furthermore, several research groups have reported a positive role of probiotics in enhancing the immune response by inducing cytokine production, such as interleukin-6 and interleukin-12 [26], as well as TNF-alpha secretion [27]. Recently, a research group reported that lipoteichoic acid from *L*. *plantarum* suppressed the production of proinflammatory cytokines and nitric oxide in LPS-stimulated cells that had infiltrated the atherosclerotic plaque in mice [28]. Conversely, *A*. *actinomycetemcomitans* produced immunosuppressive factors that were capable of impairing the human lymphocyte function by distracting cell cycle progression [29]. Thus, probiotics have several advantages that make them the promising candidates against *A*. *actinomycetemcomitans*. The aims of this study were to evaluate the potential of probiotic bacteria as a degrading agent against periodontal pathogenic *A*. *actinomycetemcomitans* and to elucidate the mechanisms underlying the observations made.

## Materials and Methods

### Strains and Culture Conditions

*A*. *actinomycetemcomitans* strains (smooth colony type) and probiotic strains are given in Table 1. *A*. *actinomycetemcomitans* strains and *Actinomycetes naeslundii* JCM 8349 were grown in a BHI broth (Wako) containing brain heart (8 g/L), peptic digested animal tissue (5 g/L), pancreatic digested casein (16 g/L), sodium chloride (5 g/L), 0.2% glucose (w/v), and disodium phosphate (2.5 g/L) with 1% yeast extract. The cultures were shaken at 120 rpm and incubated at 37°C and 5% CO_2_ for 48 h. Whereas for probiotic strains, all were grown in De Man, Rogosa, and Sharpe (MRS) broth supplemented with l mL/L Tween 80 (Fluka, Sigma-Aldrich) under anaerobic conditions by using anaerobic container and gas generators for anaerobic culture (Mitsubishi Gas Chemical co.Inc) at 37°C for 48 h.

**Table 1:** List of bacterial strains used in this study.

**Name**		**Source**
**Periodontal bacteria**	*A*. *actinomycetemcomitans* strains	
	Y4 (serotype b)	Kyushu Dental University, Japan
	SUNY 75 (serotype a)	Kyushu Dental University, Japan
	OMZ 534 (serotype e)	Kyushu Dental University, Japan
	*Actinomycetes naeslundii* JCM 8349	Japan Collection of Microorganism
**Probiotic strains**		
	*L*. *acidophilus* JCM 1021	Japan Collection of Microorganism
	*L*. *casei* subsp. *rhamnosus* NBRC 3831	National Biological Research Center, Japan
	*L*. *delbrueckii* subsp. *casei* JCM 1012	Japan Collection of Microorganism
	*L*. *fermentum* JCM 1137	Japan Collection of Microorganism
	*L*. *fermentum* NBRC 15885	National Biological Research Center, Japan
	*Lactococcus lactis* NBRC 12007	National Biological Research Center, Japan
	*L*. *casei* NBRC 15883	National Biological Research Center, Japan
	*Leuconostoc fructosum* NBRC 3516	National Biological Research Center, Japan
	*Leuconostoc mesenteroides* IAM 1046	Institute of Molecular and Cellular Bioscience (IAM), Japan
	*L*. *plantarum* NBRC 15891	National Biological Research Center, Japan
	*L*. *johnsonii* NBRC 13952	National Biological Research Center, Japan
	*L*. *sake* NBRC 3541	National Biological Research Center, Japan
	*L*. *paracasei* subsp. *paracasei* NBRC 3533	National Biological Research Center, Japan

### Biofilm Degradation Assay

The biofilm assay was performed following the technique of Pratt and Kolter [30], with some modifications. *A*. *actinomycetemcomitans* strains were grown overnight in BHI broth supplemented with 1% yeast extract and incubated at 37°C and 5% CO_2_. In order to develop the biofilm, the overnight cultures of *A*. *actinomycetemcomitans* strains were diluted to an optical density (OD) of 0.05 at 600 nm in fresh BHI medium supplemented with 1% yeast extract. This was to prepare the planktonic stage prior to developing a mature biofilm. A 200-μL aliquot of the suspension was assayed into each well of a 96-well flat bottom plate (Costar, Corning NY). All plates were incubated under static anaerobic conditions at 37°C for 72 h and formation of the biofilm was evaluated periodically by visual inspection. After a 72 h incubation period, a mature and tenacious biofilm had formed on the surface of the 96-well plate. In order to determine the effect of probiotic strains against the matured *A*. *actinomycetemcomitans* biofilm, the biofilm culture medium was removed using an aspirator and 200 μL of cell cultures of probiotic strains in fresh MRS broth (adjusted to OD 0.05 at 600 nm) were added directly onto the *A*. *actinomycetemcomitans* biofilm. As a negative control, *A*. *naeslundii* JCM 8349 cell cultures in fresh BHI broth (adjusted to OD 0.05 at 600 nm) were added directly onto the *A*. *actinomycetemcomitans* biofilm. All samples were incubated for another 24 h under static anaerobic conditions at 37°C prior to measuring the biofilm degradation. The percentage of biofilm degradation was calculated using following formula:

Percentage of degradation=(C−T)/C×100

Where *C* is the average absorbance per well for untreated biofilm and *T* is the average absorbance per well with the addition of probiotic cells or a cell-free supernatant.

### Crystal Violet Staining and Biofilm Quantification

Biofilm degradation was determined by 0.1% crystal violet staining, as previously described [30]. The microtiter plate with biofilm was gently washed by submerging the plate in a small container of distilled water three times. The plates were then dried by patting them on a piece of paper towel. This cleaning procedure removed any loosely attached cells or media that otherwise might be stained in the next step. In order to determine the total mass of biofilm, 200 μL of 0.1% crystal violet (w/v) were added into each well and dissolved for 30 min [31]. The plate was then gently rinsed using distilled water and allowed to air-dry in an incubator at 37°C for 15 min. The remaining biofilm with or without the addition of probiotic cells was visualized by photograph. Afterwards, the stained biofilm was dissolved over 30 min by a 200 μL addition of 95% ethanol into each well. The plates were read using an ELISA microplate reader (Tecan, Waco) at an absorbance of 492 nm.

### Biofilm Degradation by Co-Aggregation Assay

Based on our laboratory experiments, *Lactobacillus* spp. cells used in this study have poor biofilm formation on the microtiter plate (S1 Fig). Due to this, we suspect if the lactobacilli have poor adhesion or auto-aggregation ability between cells which might influence the degradation of biofilm. Thus, a biofilm degradation assay using a co-aggregation buffer was developed to assist the co-aggregation ability of the viable or dead lactobacilli cell pellets only with the pre-formed *A*. *actinomycetemcomitans* biofilm. Instead of degradation, co-aggregation between cells might promote the propagation of biofilm formation. Thus, this assay might reconfirm if biofilm degradation ability will be effected by co-aggregation activity. Briefly, probiotic strains were cultured overnight in MRS broth up to the mid-exponential growth phase (OD 1.4 at 600 nm). The cell pellets were extracted by centrifugation at 10 000 g for 15 min, washed twice, and suspended in a sterile co-aggregation buffer containing 0.05 M-Tris/HCl and 0.005 M-CaCI_2_ at a final pH of 7.0. The buffer was then used to stabilize the co-aggregation activity [32, 33] between cells by indirectly promoting a stronger cell attachment during biofilm activity. Dead probiotic cells were prepared by autoclaving the washed cells at 121°C for 20 min. Cells viability were reconfirmed by a spread plate on MRS agar. A 200 μL of viable or dead cell pellets in the co-aggregation buffer were then added into each well containing *A*. *actinomycetemcomitans* Y4 biofilm, except the control, and co-incubated for another 24 h under static anaerobic conditions at 37°C. Biofilm degradations were determined by 0.1% crystal violet staining.

### Cell Viability of Degraded Biofilm

Ideally, a degraded biofilm will cause a release of cells into the supernatant. To determine cell viability from the degraded biofilm, a 100 μL sample was collected by gently aspirating the supernatant without contacting the biofilm which had formed at the bottom of the well. The sample was serially diluted in sterile PBS and filtered using a 0.8 μm non-pyrogenic sterilized filter (Sartorius Stedim Biotech, Goettingen, Germany) to separate the *A*. *actinomycetemcomitans* Y4 cells from probiotic cells, and spread on BHI agar supplemented with 1% yeast extract. All plates were incubated at 5% CO_2_ for 48 h and colony counts were performed within the range of 30 to 300 colonies.

### pH Adjustment in Culture Supernatant

Probiotic strains were grown in MRS broth for 24 h at 37°C in an anaerobic jar to the mid-log growth phase (OD 1.4 at 600 nm). The cell-free culture supernatant was collected via centrifugation at 10 000 g for 15 min at 4°C, followed by filter sterilization using a 0.2 μm cellulose acetate filter (Sartorius Stedim, Germany). The supernatants were adjusted to pH 6.5 using 1M HCI and concentrated 10-fold by a centrifugal evaporator (VC-L5SP TAITEC). In order to evaluate the influence of a low pH of the culture supernatant of probiotic strains on biofilm degradation, the pH was adjusted to 6.5 and this cell-free supernatants were added to wells containing biofilm. Supernatants without a pH adjustment were used as a control. All samples were incubated under static anaerobic conditions at 37°C for 24 h. Activities associated with the adjusted pH and control were compared. pH was measured by a LAQUAtwin compact pH meter (Horiba Ltd, Kyoto, Japan).

### Effect of Lactic Acid on Biofilm Degradation

The concentration of lactic acid was quantified because it was one of the main compounds produced by probiotic strains in this study. After 24 h incubation following the addition of probiotic cells, the spent supernatant was collected and analyzed for its lactic acid concentration by high performance liquid chromatography (HPLC, Shimadzu LC-10AD) [34]). The range of lactic acid concentrations produced was used as a reference to determine the effect of exogenously added lactic acid on biofilm degradation. The biofilm assay was performed as previously described. On incubation day three, 200 μL of lactic acid at final concentrations of 100, 150, 200 and 250 mM in BHI broth were added to wells containing *A*. *actinomycetemcomitans* Y4 biofilm, except the control well. The plate was incubated at 37°C under anaerobic conditions for 24 h prior to biofilm measurements.

### Effect of Enzymes on Biofilm Degradation

To investigate the factors that contribute to biofilm degradation, we performed an enzyme treatment on the formed biofilm. Enzymatic solutions with a final concentration of 5 μg/mL of proteinase K (Nacalai Tesque), α-amylase (Wako Pure Chemical Industries Co. Ltd.), or lipase (Nacalai Tesque) in Tris-HCI buffer pH 7.0 were prepared. Each enzymatic solution was then added to wells containing biofilm, as a single or combination enzyme mixture and incubated under static anaerobic conditions at 37°C for 24 h. Biofilm measurements were performed as previously described. Biofilm without this treatment was used as a control.

### Effect of Lipase Inhibitor on Biofilm Degradation

The potent degradation effect by the lipase enzymes was reconfirmed by using a lipase inhibitor (Tetrahydrolipstatin,Nacalai Tesque). Briefly, a stock solution of lipase inhibitor was prepared in 50% DMSO. Subsequently, the solution was diluted to 250 μg/mL using sterilized distilled water and then mixed with each probiotic cell-free supernatant, with the final concentration of 5 μg/mL. Subsequently, each supernatant with or without the lipase inhibitor was added into wells having the biofilm of *A*. *actinomycetemcomitans* strains, and incubated anaerobically under a static condition at 37°C for 24 h. As a negative control, *A*. *naeslundii* JCM 8349 cell-free supernatant was used and tested with or without the lipase inhibitor. Biofilm measurements were performed as previously described.

### Lipase Assay

To determine the lipase activity profile of probiotic cells and *A*. *naeslundii* JCM 8349 an overnight culture supernatants of those bacteria were collected by centrifugation at 10 000 g for 10 min at 4°C, filter sterilized using a 0.2 μm cellulose acetate filter and applied to a lipase assay using a Lipase kit S (DS Pharma Biomedical), following the manufacturer’s instructions. A lipase enzyme level of 0.01 mg/mL was used as a positive control, and sterilized distilled water was used as negative control.

### Statistical Analysis

Data from at least three independent experiments represented as mean ± SD (standard deviation). Comparisons were performed by the means of Student t-test using GraphPad Software and P ˂ 0.05 was considered as significant.

## Results

### Biofilm Degradation by Probiotic Bacteria

We investigated the potential of probiotic bacteria to degrade the formed biofilm of a periodontal pathogen *A*. *actinomycetemcomitans in vitro*. Application of nutrient rich medium as medium growth for biofilm assay facilitates the optimum growth of the probiotic cells and produces active enzymes that may be contribute to the biofilm degradation.

**Fig 1:**
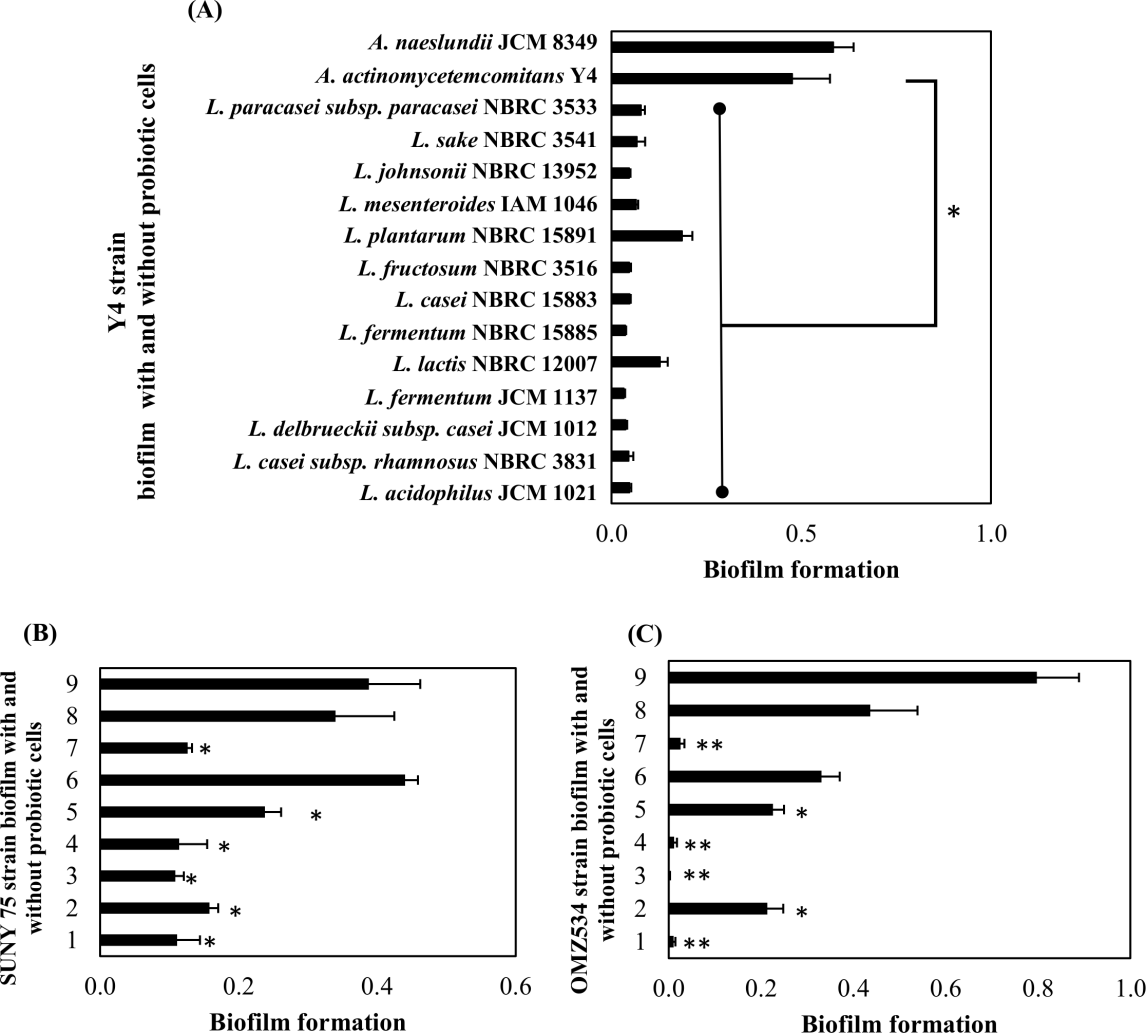
Biofilm values of *A*. *actinomycetemcomitans* strains with addition of probiotic bacteria on pre-formed biofilm. (A) represents Y4 strain (serotype b). Whereas (B and C) represents SUNY75 strain (serotype a) and OMZ534 strain (serotype e) respectively. Probiotic species and controls were labelled with number as follows; 1: *L*. *fermentum* JCM 1137, 2: *L*. *acidophilus* JCM 1021, 3: *L*. *fermentum* NBRC 15885, 4: *L*. *fructosum* NBRC 3516, 5: *L*. *plantarum* NBRC 15891, 6: *L*. *casei subsp*. *rhamnosus* NBRC 3831, 7: *L*. *johnsonii* NBRC 13952. Positive controls (8) are *A*. *actinomycetemcomitans* biofilm without probiotic addition, and *A*. *naeslundii* JCM 8349 was used as a negative control (9). Bars represent the mean, error bars represent standard deviation and significance was measured using paired T-test (* = P< 0.05, ** = P< 0.001).

Interestingly, after 24 h incubation, a significant biofilm degrading activity was observed for all the probiotic strains against the Y4 strain, with a different ratio of degradation (Fig 1A). Six of the seven probiotic strains showed a significant biofilm degradation against SUNY 75 (Fig 1B). In addition, for OMZ 534, four of the seven probiotic strains showed a high significant ability of biofilm degradation (Fig 1C). The probiotic strains did not have a big effect against the biofilm of SUNY 75 strain compared to Y4 and OMZ 534 strains in which more than 90% of the biofilm was degraded by several probiotic strains. The range of biofilm degradation for SUNY 75 was approximately 18 to 42% (Table 2).

**Table 2:** Degradation percentage after the addition of probiotic cells on pre-formed *A*. *actinomycetemcomitans* strains.

**Potential Probiotic strains**	Percentage of *A*. *actinomycetemcomitans* biofilm degradation (%)		
	**Y4**	**SUNY 75**	**OMZ 534**
*L*. *fermentum* JCM 1137	93 ± 1	65 ± 13	97 ± 1
*L*. *delbrueckii* subsp. *casei* JCM 1012	92 ± 1	N/A	N/A
*L*. *fermentum* NBRC 15885	92 ± 1	65 ± 12	99 ± 0
*L*. *casei subsp*. *rhamnosus* NBRC 3831	90 ± 3	-29 ± 5	24 ± 10
*L*. *johnsonii* NBRC 13952	90 ± 1	60 ± 11	94 ± 2
*L*. *fructosum* NBRC 3516	90 ± 1	65 ± 11	97 ± 1
*L*. *acidophilus* JCM 1021	90 ± 1	53 ± 3	51 ± 8
*L*. *casei* NBRC 15883	90 ± 1	N/A	N/A
*L*. *sake* NBRC 3541	86 ± 5	N/A	N/A
*L*. *mesenteroides* IAM 1046	86 ± 4	N/A	N/A
*L*. *paracasei subsp*. *paracasei* NBRC 3533	84 ± 2	N/A	N/A
*L*. *lactis* NBRC 12007	73 ± 4	N/A	N/A
*L*. *plantarum* NBRC 15891	61 ± 6	30 ± 6	48 ± 5

Y4 (serotype b), SUNY 75 (serotype a) and OMZ 534 (serotype e) strains of *A*. *actinomycetemcomitans* were used for this study. The ± is referring to standard deviation (SD) from at least three independent experiments. NA represent not available data from that particular strains.

### Degradation Ability of Probiotic Bacteria Cells and Supernatant

**Fig 2:**
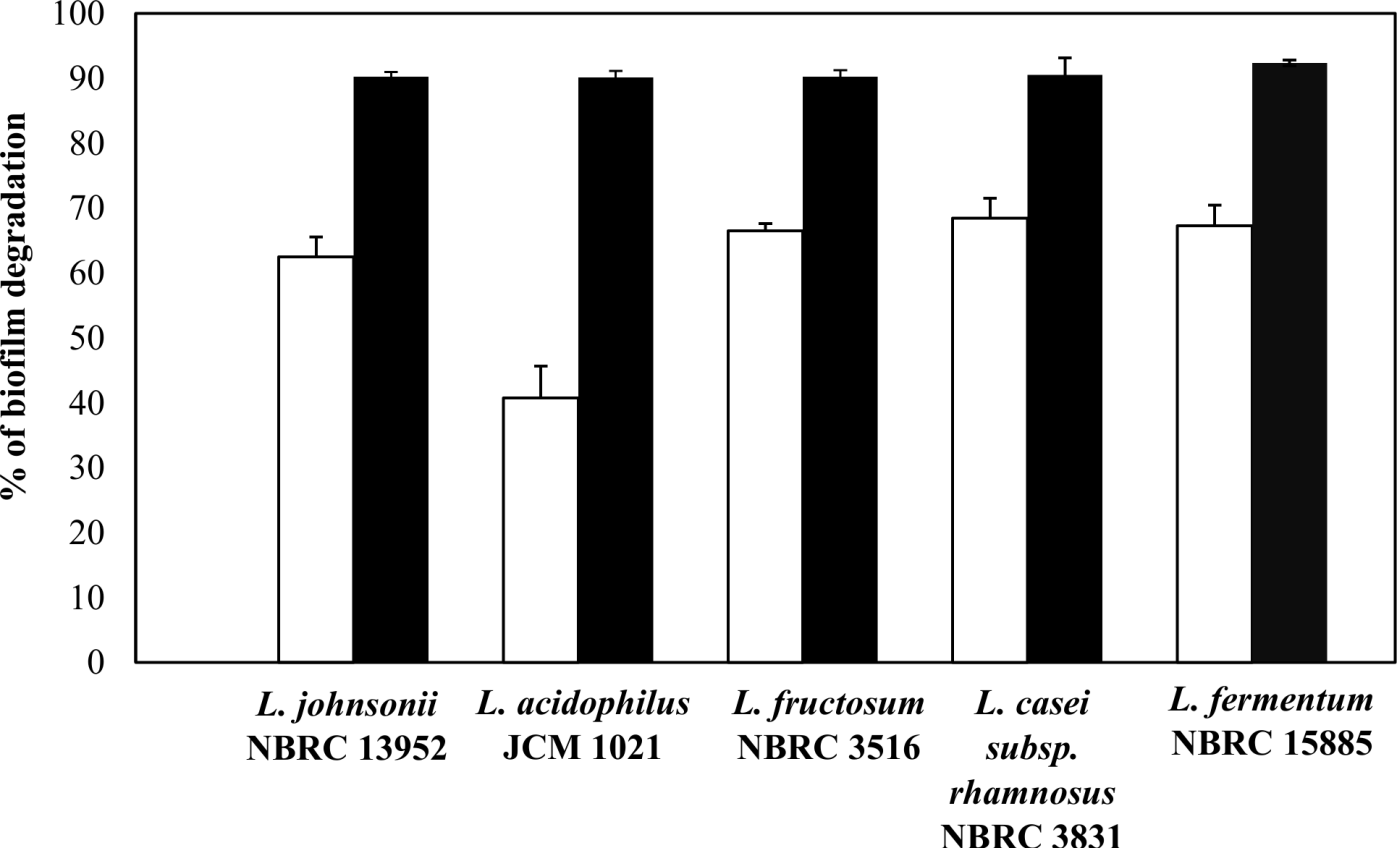
Percentage of biofilm degradation by probiotic cells or cell-free supernatant (concentrated) against *A*. *actinomycetemcomitans* Y4 biofilm. Black bars represent biofilm degradation by cells and white columns represent biofilm degradation by a cell-free supernatant. Bars represent the mean, error bars represent standard deviation.

Regardless of the species, all probiotic bacteria demonstrated their ability to degrade *A*. *actinomycetemcomitans* Y4 biofilm. Five probiotic species were randomly selected to determine whether biofilm degradation was driven by the cells or extracellular compounds produced in the supernatant. We observed that both the concentrated supernatant and cells promoted the biofilm degradation activity at different levels of intensity (Fig 2). Interestingly, the probiotic cells showed higher biofilm degradation activity compared with a 10 times concentrated cell-free supernatants. The phenomena gain our interest to explore the potential of the cells and what is the possible factors contributing to the biofilm degradation.

### Probiotic Cell Pellet and Degradation Activity

The influence of probiotic cell pellets on the degradation activity was reconfirmed by a coaggregation assay. This assay might facilitate direct cell attachment between the probiotic cells and the biofilm, without the influence of nutrients or the low pH of the culture medium. The co-aggregation buffer may also promote and stabilize the aggregation activity that occurs between cells.

**Fig 3:**
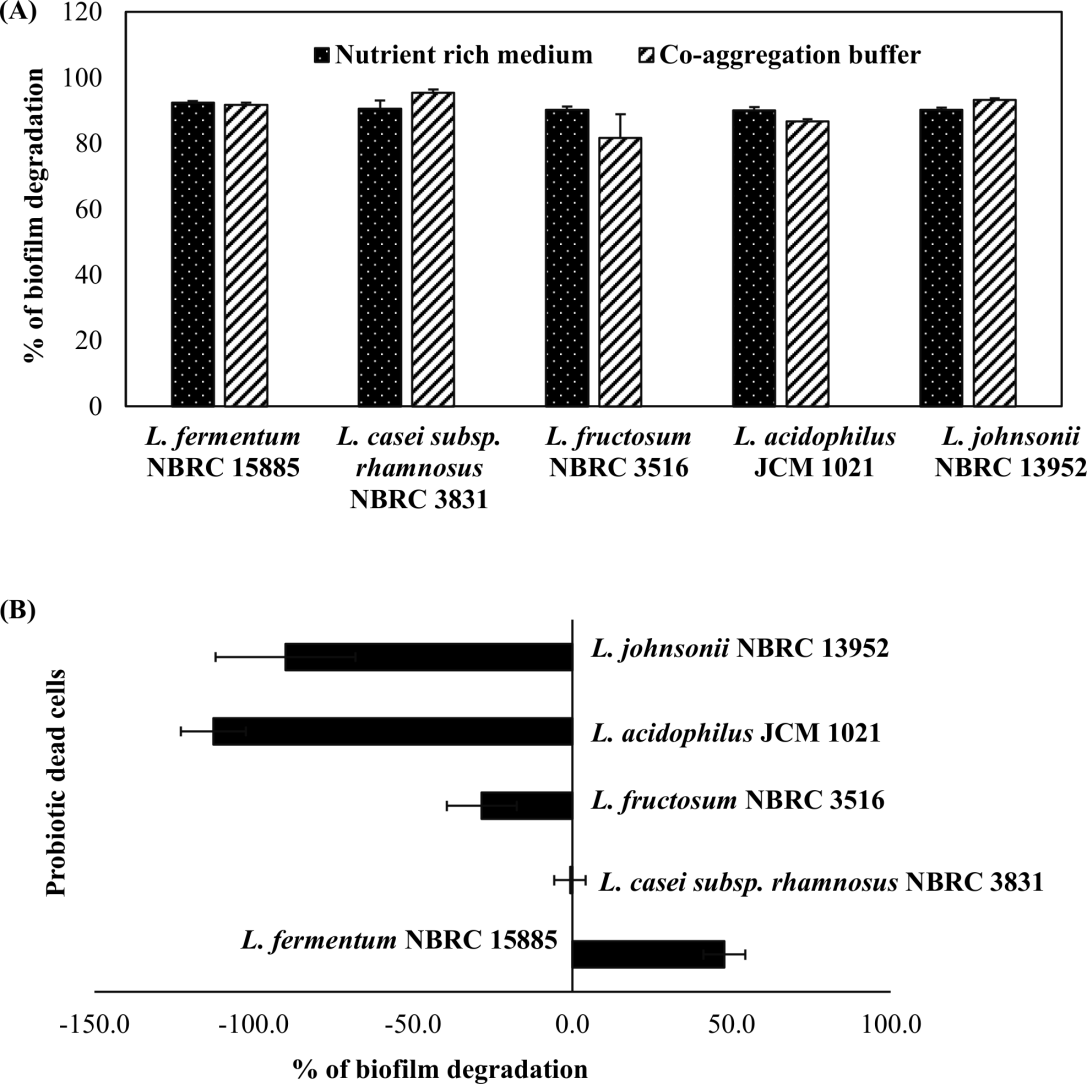
Influence of culture medium nutrient and cell viability on biofilm degradation activity. Fig 3A represents a comparison of biofilm degradation by probiotic cells in a nutrient rich medium and a co-aggregation buffer. The nutrient rich medium contained a low density of probiotic cells (OD 0.05 at 600 nm absorbance). Washed cell pellets from an overnight culture were co-incubated with pre-formed *A*. *actinomycetemcomitans* Y4 biofilm to form the co-aggregation buffer. Fig 3B represents biofilm degradation by dead probiotic cells (autoclaved). Bars represent the mean, error bars represent standard deviation.

In this assay, the *A*. *actinomycetemcomitans* Y4 biofilm was propagated to be two-fold higher (biofilm value: 1.04) than in the BHI medium (0.48). Corresponding this, the percent biofilm degradation by cell pellets was shown similar for cells in a nutrient rich medium (MRS) or co-aggregation buffer (Fig 3A). This is an important finding as it demonstrates that probiotic cells have an effective and continual impact against formed biofilm. The potential of cell pellets was further investigated using dead probiotic cells as a degrading agent. Our findings showed that only dead cells of *L*. *fermentum* NBRC 15885 had the ability to affect the pre-biofilm, at half the rate compared to viable cells. Conversely, other strains of dead probiotic cells lost their ability to degrade the biofilm. In spite of the degradation, dead cells of *L*. *johnsonii* NBRC 13952, *L*. *acidophilus* JCM 1021, and *L*. *fructosum* NBRC 3516 did propagate in the biofilm and contributed to the degradation values (Fig 3B). These results indicate that a factor for the biofilm degradation should be produced by the viable probiotic strains.

### Viability of *A*. *actinomycetemcomitans* Y4 from Degraded Biofilm

**Fig 4:**
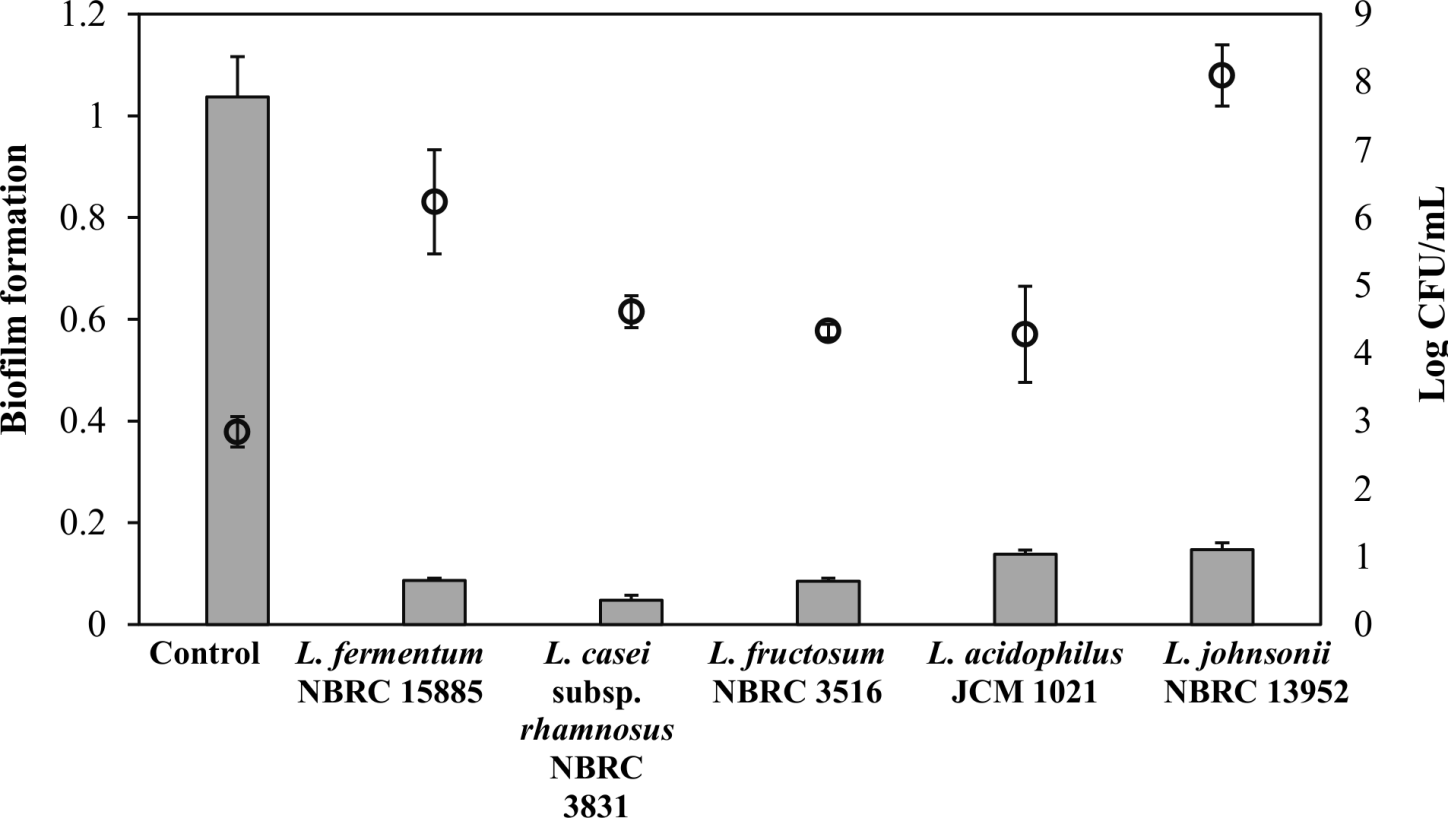
Co-incubation of pre-formed *A*. *actinomycetemcomitans* Y4 biofilms with probiotic cells leading to biofilm degradation. *A*. *actinomycetemcomitans* Y4 biofilm was allowed to form under static anaerobic conditions for 72 h before a further 24 h co-culture with probiotic cells in a co-aggregation buffer. Biofilm with the addition of a co-aggregation buffer was used as a control. Grey columns represent biofilm formation and empty circles represent viable *A*. *actinomycetemcomitans* Y4 from the biofilm supernatant. Bars and circle represent the mean, error bars represent standard deviation.

In theory, degraded biofilm dispersed as live or dead cells into the culture medium. Therefore, viability of the degraded biofilm following the addition of probiotic cells was assessed. As expected, the degraded biofilm showed a higher cell number compared with the control (Fig 4). However, the number of viable bacteria in the culture supernatant was not proportional to the percentage of biofilm degradation. An existence of a high number of viable cells from a biofilm assay with *L*. *fermentum* NBRC 15885 and *L*. *johnsonii* 13952 might suggest that the degradation activity was possibly not due to an inhibitory or killing effect of the probiotic cells against *A*. *actinomycetemcomitans* Y4.

### Effect of Low pH and Lactic Acid on Biofilm Degradation

**Fig 5:**
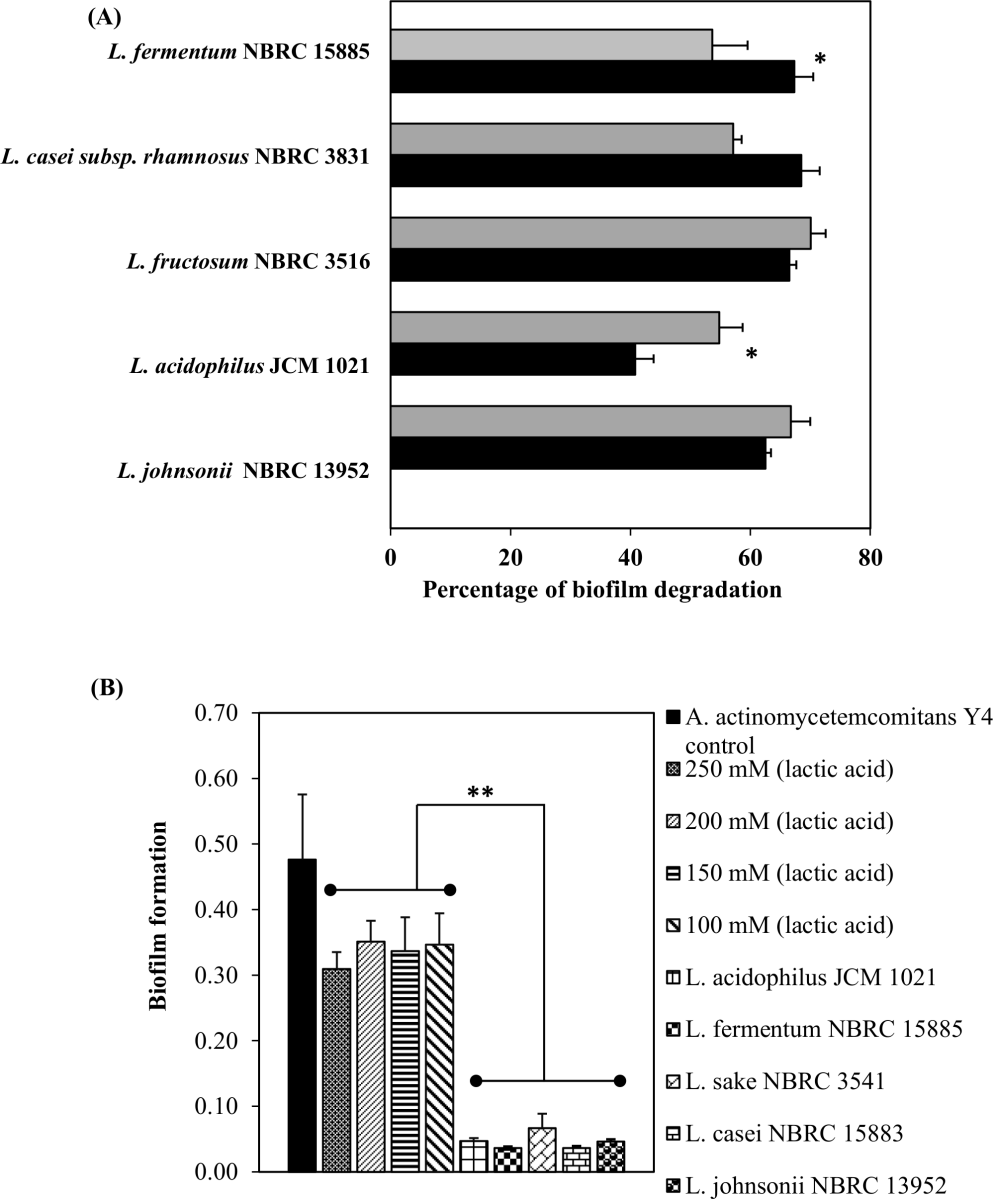
Influence of probiotic bacteria supernatant and lactic acid on biofilm growth. Percent biofilm degradation following (A) the addition of an untreated cell-free supernatant (black column) or an adjusted pH (6.5) cell-free supernatant (gray column). (B) Biofilm growth of *A*. *actinomycetemcomitans* Y4 with lactic acid at various concentrations compared with the addition of probiotic cells. Biofilm of *A*. *actinomycetemcomitans* Y4 was allowed to pre-form under static anaerobic conditions for 72 h prior to the addition of lactic acid or probiotic cells. Bars represent the mean, error bars represent standard deviation and significance was measured using paired T-test (* = P< 0.05, ** = P< 0.001).

Any of the probiotic species used in this study could have contributed to a low pH of the culture supernatant due to metabolic activity and production of organic acids. To assess whether a low pH had contributed to biofilm degradation, the activity was confirmed using an adjusted pH (6.5) cell-free supernatant. Initial pH range of the untreated cell-free culture supernatant was from pH 4.4 to pH 5.2. We observed that the percent biofilm degradation was relatively the same between the untreated and adjusted pH cell-free supernatant (Fig 5A). Except for *L*. *fermentum* NBRC 15885 and *L*. *acidophilus* JCM 1021 which show a significant different after pH changes. This result suggests that a low pH condition did not contribute to the degradation activity of the biofilm.

Because lactic acid is one of potent metabolites produced by probiotic species, effect of lactic acid was determined using the same method. The concentrations of lactic acid used ranged from 100 to 250 mM (S2 Fig), which might represent the same amount produced by probiotics in the actual biofilm assay. All probiotic cells significantly caused a higher degradation activity compared to lactic acid (Fig 5B). Lower values of biofilm formation compared to the *A*. *actinomycetemcomitans* Y4 control indicated that a higher degradation activity had occurred. This finding indicates lactic acid has vice-versa effect toward biofilm degradation. Thus, other factors have roles the biofilm degradation activity.

### Disruption of Biofilm Growth by Enzymes Activity

**Fig 6:**
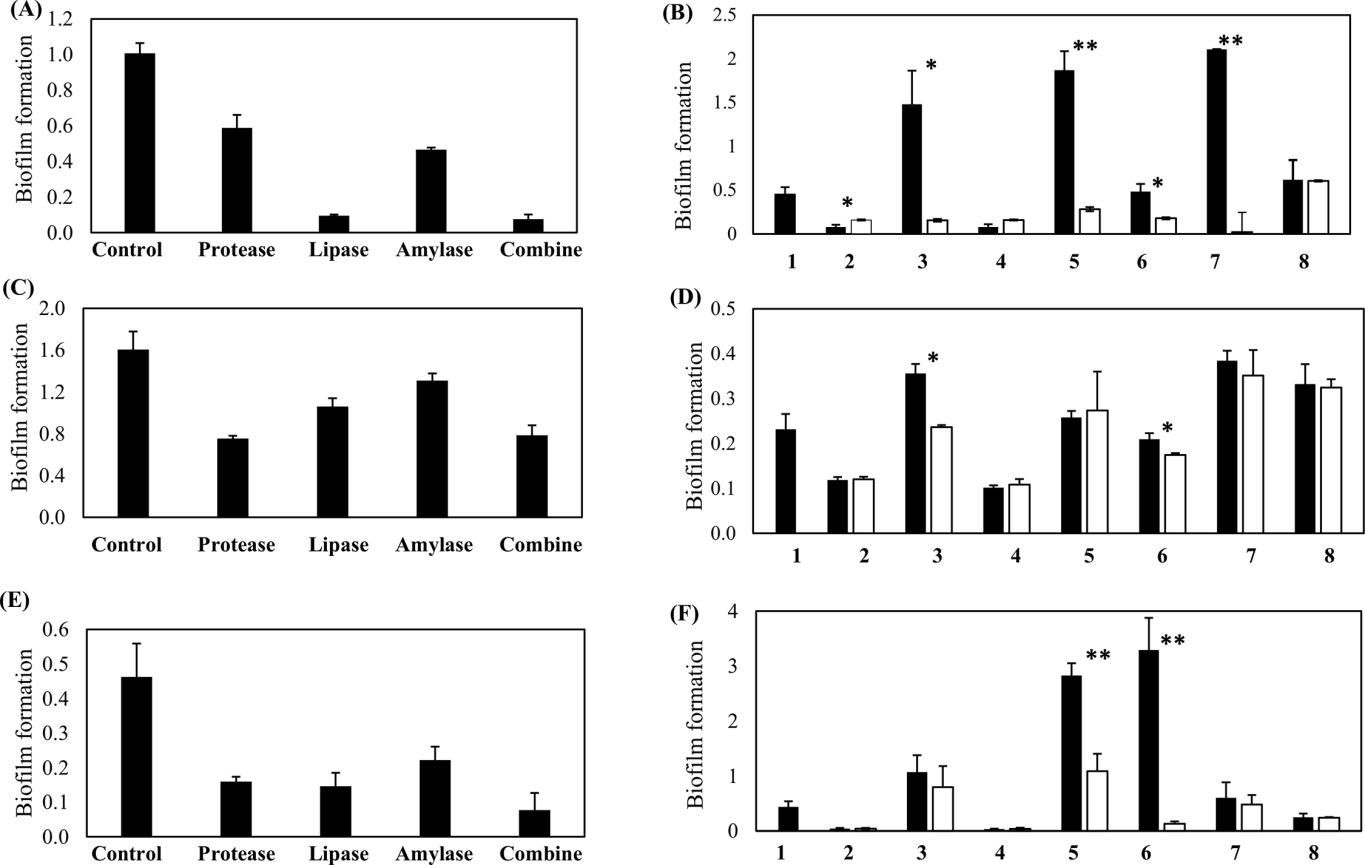
**Effect of enzymes and influence of lipase inhibitor on the formed biofilm** (A, C and E) show degradation activity by the presence of proteinase K, amylase, lipase, or a combination of all enzymes against Y4, SUNY 75, and OMZ 534 respectively. (B, D and F) are the influence of lipase inhibitor on biofilm degradation against Y4, SUNY 75 and OMZ 534 respectively. Probiotic strains and controls were labelled as follows. 1: Positive control (*A*. *actinomycetemcomitans* biofilm without the addition of probiotic cells), 2: *L*. *fermentum* NBRC 15885, 3: *L*. *casei* subsp. *rhamnosus* NBRC 3831, 4: *L*. *fructosum* NBRC 3516, 5: *L*. *acidophilus* JCM 1021, 6: *L*. *johnsonii* NBRC 13952, 7: *L*. *plantarum* NBRC 15891, 8: *A*. *naeslundii* JCM 8439 (negative control species). Bars represent the mean, error bars represent standard deviation and significance was measured using paired T-test (* = P< 0.05, ** = P< 0.001).

The bacterial biofilm matrix contains proteins, nucleic acid, polysaccharides, lipids, mineral ions, and cell debris [35]. To examine the effect of enzymes on biofilm activity, proteinase, amylase, lipase, and all enzymes in combination were applied to the pre-formed biofilm using the same method for the addition of probiotic cells. Our findings showed that all enzymes have a degrading effect against the *A*. *actinomycetemcomitans* Y4 biofilm (Fig 6A). A potent degradation effect was demonstrated by lipase and enzymes in combination with a 90.5% and 92.4% reduction of biofilm, respectively. Proteinase K and amylase showed a moderate effect with a 41.2% and 53.6% reduction of biofilm, respectively. The influence of lipase enzyme on biofilm degradation in Y4 strain was reconfirmed by adding a lipase inhibitor to the probiotic cell-free supernatant (Fig 6B). The activity of biofilm degradation significantly reduced by the addition of the lipase inhibitor into the probiotic cell-free supernatants (Fig 6B). This might suggest that lipase plays an important role in the biofilm degradation of Y4 strain. In contrast, *L*. *fermentum* NBRC 15885 and *L*. *fructosum* NBRC 3516 showed no influence for biofilm degradation in the presence of the lipase inhibitor, meaning that the biofilm degradation by these bacteria may be due to another mechanism. *A*. *naeslundii* JCM 8349 (as a negative control) showed no difference with or without the lipase inhibitor.

The biofilm of the other serotype of *A*. *actinomycetemcomitans* SUNY 75 was moderately affected by the three enzymes (protease, lipase, and amylase), of which the highest biofilm degradation activity was shown in the presence of protease (Fig 6C). The addition of lipase inhibitor to the probiotic cell-free supernatant showed an inhibitory effect for the biofilm degradation by two probiotic strains, *L*. *casei* subsp. *rhamnosus* NBRC 3831 and *L*. *johnsonii* NBRC 13952; however, no/less effect was observed for other potential probiotics (Fig 6D). On the other hand, the OMZ 534 biofilm was greatly affected by the three enzymes as shown in a result that more than 50% of biofilm was degraded by protease, lipase, or amylase (Fig 6E). In addition, the biofilm degradation by *L*. *acidophilus* JCM 1021 and *L*. *johnsonii* NBRC 13952 was remarkably inhibited by a lipase inhibitor (Fig 6F). Other probiotic strains also showed an inhibitory effect of biofilm degradation but the effect was not significant in conditions with or without the lipase inhibitor. *A*. *naeslundii* JCM 8349 (used as a negative control) showed no difference with or without the lipase inhibitor.

**Fig 7:**
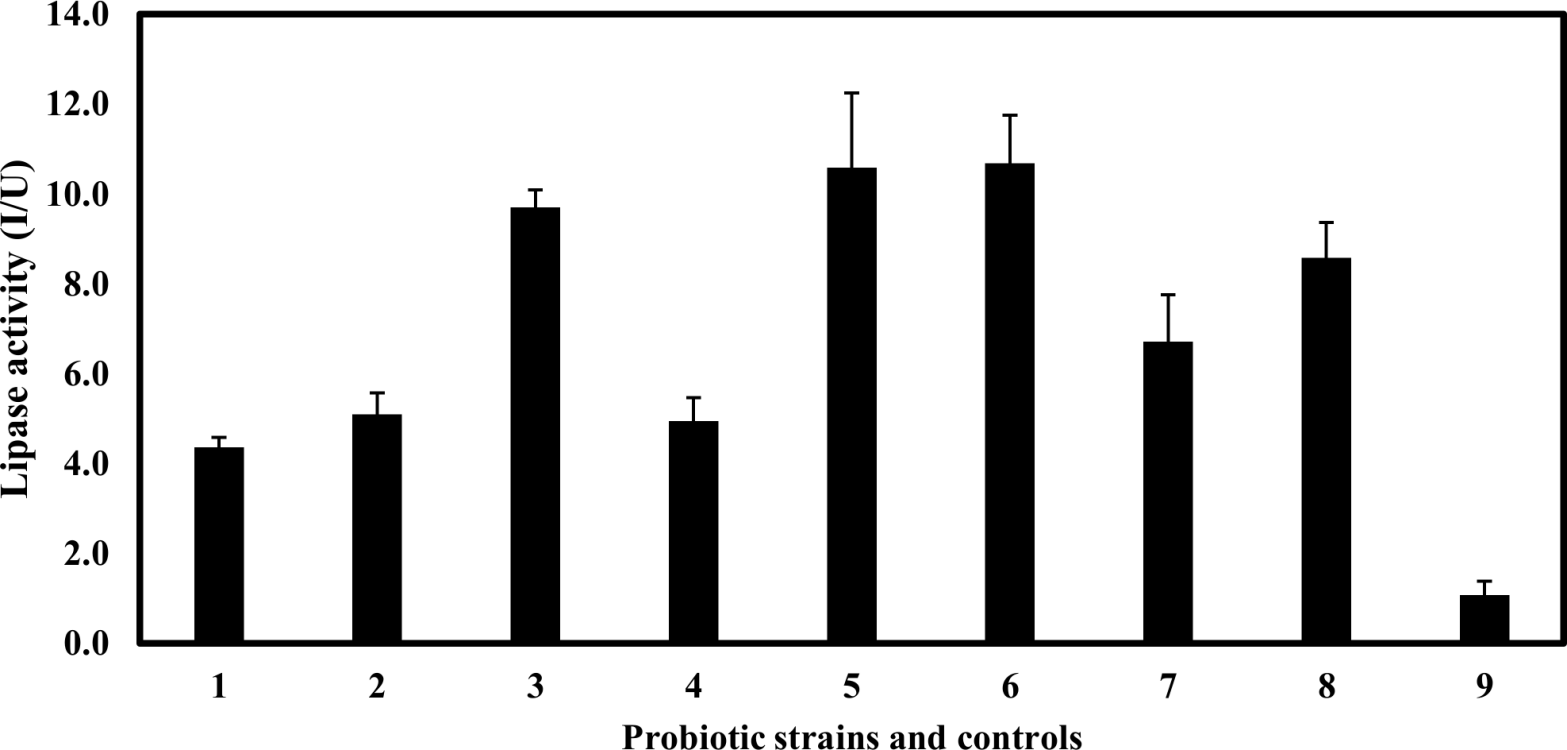
Profile of lipase activity from an overnight culture in a supernatant of probiotic bacteria. Probiotic strains and controls were labelled was follows. 1: Positive control (lipase enzyme with final concentration 0.01 mg/mL), 2: *L*. *fermentum* NBRC 15885, 3: L. *casei* subsp. *rhamnosus* NBRC 3831, 4: *L*. *fructosum* NBRC 3516, 5: *L*. *acidophilus* JCM 1021, 6: *L*. *johnsonii* NBRC 13952, 7: *L*. *plantarum* NBRC 15891, 8: *A*. *naeslundii* JCM 8439 and 9: negative control (sterilized distilled water). Bars represent the mean, error bars represent standard deviation.

In order to verify the importance of lipase in the biofilm degradation, lipase activities in all the strains used in this study were measured. As a result, the lipase activities of all probiotic strains cell-free supernatants were higher than the positive control (Fig 7). The relatively-high lipase activities were detected from the supernatants of *L*. *acidophilus* JCM 1021, L. *johnsonii* 13952, and *L*. *casei* subsp. *rhamnosus* NBRC 3831. In addition, although the negative control, *A*. *naeslundii* JCM 8349 did not have the ability of biofilm degradation, a high lipase activity was detected from the strain. We hypothesize that lipase enzymes derived from the probiotic strains may be highly specific to the biofilm of *A*. *actinomycetemcomitans* strains due to the substrate specificity as there have been some literatures that describe the diversity of lipase enzymes [36–38]. Furthermore, the biofilm degradation by *L*. *fermentum* NBRC 15885 and *L*. *fructosum* NBRC 3516 might be due to another mechanism because they did not have a high lipase activity despite the cell-free supernatants from both species had great biofilm degradation abilities for *A*. *actinomycetemcomitans* strains tested.

## Discussion

A biofilm is a group of bacteria which stick together, adhere to surfaces, are phenotypically resistant, and very difficult to eradicate from a living host. Various diseases are initiated by or are associated with biofilm formation such as cystic fibrosis, otitis media, and chronic prostatitis [39]. Diseases specific to *A*. *actinomycetemcomitans* are infective endocarditis and periodontitis [9, 13, 40].

Antibiotic applications to counteract these infections are not promising because their high bacterial load facilitates antibiotic resistance. During scaling, root planning, or periodontal surgery, the administration of antibiotics is to ensure that *A*. *actinomycetemcomitans* is eliminated from periodontal lesions. Unfortunately, at the biofilm stage, almost all species are protected by their EPS thus, reducing the effectiveness of antibiotics. For example, ampicillin and cephalexin have been shown to inhibit *A*. *actinomycetemcomitans* Y4 biofilm formation during the first 24 h of incubation, but an inverse effect was observed for matured biofilm at 48 h incubation with a significant increase in adenosine triphosphate levels [14]. This phenomenon indirectly demonstrates the continuous challenges faced when treating an infection. Various studies have discussed the issue of how to tackle biofilm formation by pathogens. Several of them highlighted the promising effect of probiotic strains including *L*. *rhamnosus*, *L*. *salivarius*, *L*. *reuteri*, and *W*. *cibaria* as potential candidates for the treatment of oral diseases by suppressing the growth of periodontal pathogens [41–44]. Probiotic bacteria are a good alternative due to several advantages that these organisms have that are believed to counteract pathogenesis by periodontal pathogens. Furthermore, their ability to induce an immunomodulatory response by an increase in cytokine production [45], an antiviral response against vescular stomatitis via interaction with macrophages [46], and induction of nitric oxide synthesis [47] might provide a powerful effect against pathogens which virulence toward immune cells, as previously reported for *A*. *actinomycetemcomitans* [6, 48–50].

Using 13 species of probiotic bacteria, one valuable finding in this study was their high degradation activity against an *A*. *actinomycetemcomitans* biofilm. Eight of the 13 strains and four of the seven strains had a more than 90% biofilm degradation efficiency against the strains, Y4 and OMZ 534, respectively. *A*. *naeslundii* JCM 8349 which was used as a negative control strain is implicating in various tooth cavities [51, 52] and the results indicate that potential probiotic bacteria have surely a great ability of biofilm degradation. Effective biofilm degradation by probiotic cells directly to the pre-formed biofilm might suggest another novel mechanism. However, given that the determination of biofilm degradation using the crystal violet assay does present certain limitations (for example, it does not give a measure of biofilm viability as it stains both live and dead bacteria cells, EPS, and extracellular DNA), assessment of the relative proportion of dead and living biofilm cells might provide useful information regarding the mechanism underlying the observed effects.

An overnight incubation of *A*. *actinomycetemcomitans* Y4 biofilm (control) in a co-aggregation buffer showed an approximate 2-fold higher propagation than in a nutrient-rich medium. A co-aggregation buffer might induce a higher auto-aggregation between cells which later assembled into a bigger biofilm. In addition, environmental and pH changes might also influence *A*. *actinomycetemcomitans* biofilm formation [53]. Otherwise, an addition of probiotic cells and co-aggregation of those cells with the biofilm would be expected to contribute to a higher biofilm. However, the degradation of biofilm activity was robust and there was almost no difference between the co-aggregation buffer and the nutrient-rich medium. This suggests that the activity was not nutrient-dependent and that direct cell contact was possibly a physical factor that contributed to biofilm degradation. In theory, a co-aggregation between cells will contribute to a higher biofilm formation [54, 55]. This phenomenon was observed with dead probiotic cells against the pre-formed biofilm. A negative biofilm degradation activity associated with dead probiotic cells indicated that viable cells were potent agents and that active compounds were possibly produced by the viable cells. A reduced biofilm showed that the addition of probiotics onto the biofilm might suggest a low co-aggregation activity between probiotic cells and the biofilm. The higher number of viable cells detected in the supernatant of degraded biofilm compared to the control proves that there was a disassociation between the cells in the biofilm. Because the number of viable cells was not correlated with the rate of biofilm degradation, various factors such as strain-specific inhibitory or non-inhibitory effects might also play a role. *A*. *actinomycetemcomitans* biofilm contains an assemblage of cells which are extremely tenacious on surfaces [1] and are resistant to removal agents such as detergents, proteases, heat, sonication, and vortex agitation [56]. Biofilms are always enclosed in a complex matrix which is primarily structured by microbial cells and extracellular polymeric substances (EPS) [39]. The composition and structure of EPS varies widely among bacterial species [18]. In addition, established biofilms have been reported to be variably susceptible against enzymatic treatments. For example, protease, amylase, and pectinase enzymes from *Aspergillus clavatus* degrades the biofilms of *Pseudomonas aeruginosa*, *Bacillus subtilis*, and *Staphylococcus aureus* [35]. Another study reported that trypsin significantly reduced biofilm formation and, by contrast, proteinase K enhanced biofilm formation of a *Rhodococcus ruber* C208 biofilm [57]. Our findings demonstrate that the lipase enzyme and the mixed enzyme of lipase, protease, and amylase provided a powerful degradation biofilm activity against the *A*. *actinomycetemcomitans* Y4 and OMZ 534 strain, whereas partially in SUNY 75. Lipase activities with the potency of biofilm degradation ability against the Y4 shows a certain relationship between the *L*. *casei* subsp. *rhamnosus* NBRC 3831, *L*. *acidophilus* JCM 1021, *L*. *johnsonii* NBRC 13952, and *L*. *plantarum* NBRC 15891. However, the relationship is not highly corresponded to the other serotypes, SUNY 75 and OMZ 534 which showed no/less effect of biofilm degradation in the presence of lipase enzyme. Variations in the effect of enzymes for the biofilm degradation were observed in *A*. *actinomycetemcomitans* strains (three serotypes) because *A*. *actinomycetemcomitans* strains are with the large genetic variations [58–61], by which the property of each *A*. *actinomycetemcomitans* strain can be diverse. In fact, other factors such as LPS and EPS components which are varied between *A*. *actinomycetemcomitans* serotypes [62, 63] may be influential in the degradation of biofilm [64]. The genetic variation of *A*. *actinomycetemcomitans* may be one of the reasons why the biofilm degradation of SUNY 75 was harder than that of other serotypes. Also, instead of lipase enzyme, in SUNY 75 the treatment by proteases showed the highest biofilm degradation activity among the three enzymes (protease, lipase, and amylase), indicating that the composition of biofilm in SUNY 75 may be different with the other serotypes of *A*. *actinomycetemcomitans* strains. In addition, *L*. *fermentum* NBRC 15885 and *L*. *fructosum* NBRC 3516 which have a low lipase activity, able to sustain their biofilm degradation ability against Y4 and OMZ 534, might have another mechanism of biofilm degradation independent of the lipase activity. Thus, the biofilm degradation using potential probiotics may have a variety of strategies because *A*. *actinomycetemcomitans* strains are with the large genetic variations [58–61]. There is a possibility that the high biofilm degradation effect by the lipase enzyme was due to the digested lipoprotein in the *A*. *actinomycetemcomitans* Y4 biofilm matrix. Paul-Satyaseela et al. [65] reported that the outer-membrane proteins of *A*. *actinomycetemcomitans* contained peptidoglycan associated lipoprotein, which has a strong immunoreactivity. This was also supported by another study which identified the proteins from an *A*. *actinomycetemcomitans* strain D7S biofilm by LC-MS/MS [66]. Their findings showed that a relatively high abundance of the protein predicted that it was either periplasmic or located in the outer membrane. In an analysis of extracellular proteins of a single strain, it was found that 250 proteins were grouped into lipoproteins and outer membrane proteins [65]. Another study researched the amyloid-like fiber formation in rough and smooth phenotypes of *A*. *actinomycetemcomitans* strains [67] using Congo red (CR) as a binding assay, and confirmed that this species binds to CR. Congo red is a hydrophobic diazo dye which binds to lipids, lipoproteins, and a variety of amyloid proteins [67]. Amyloid-like fibers are abundant in natural biofilms [67] and are described as highly organized protein aggregates which are resistant to chemical or temperature denaturation and proteases digestion [68]. In the recent study by Chalabaev et al. (52), it was reported that the biofilm of gram-negative bacteria was associated with an increased level of lipid A palmitoylation, which influenced their antimicrobial resistance and inflammatory response. An abundance of lipids or lipoproteins reported by previous studies support the role of the lipase enzyme as a plausible key factor in biofilm degradation.

In conclusion, probiotic bacteria demonstrate a robust degradation activity on *A*. *actinomycetemcomitans* Y4 and OMZ 534 strain, and a moderate effect against SUNY 75 strain. Lipase enzyme from probiotic strains might be an influential factor in the biofilm degradation against *A*. *actinomycetemcomitans* Y4 and OMZ 534 strains. This novel property can be utilized as a starting point on the usage of probiotic cells as agents that can directly interact with biofilms at a clinical level. However, further research and more specific analyses need to be conducted in order to exemplify the mechanisms underlying this activity. Contrasting roles of probiotic bacteria and the periodontal pathogen towards host cells contribute to promising techniques to control infections *in vivo*.

## Supporting Information

S1 Fig Biofilm formation of probiotic strains and *A*. *actinomycetemcomitans* Y4 in mono-culture. Initial OD of each sample of cell culture suspensions were 0.05 at 600nm. All samples were incubated in anaerobic condition at 37°C for 24 hour. Bars represent the mean and error bars represent standard deviation.
(TIFF)

S2 Fig Amount of lactic acid produced in biofilm assay, 24 hour after probiotic cell culture addition. Numbers represent strains as follows; 1: *Lactococcus lactic* NBRC 12007, 2: *L*. *johnsonii* NBRC 13952, 3: *L*. *casei subsp*. *rhamnosus* NBRC 3831, 4: *Lactobacillus paracasei subsp paracasei* 3533, 5: *Leuconostoc mesenteroides* IAM 1046, 6: *L*. *sake* NBRC 3541, 7: *L*. *fermentum* NBRC 15885, 8: *L*. *casei* NBRC 15883, 9: *L*. *plantarum* NBRC 15891 and 10: *Leuconostuc fructosum* NBRC 3516. Bars represent the mean and error bars represent standard deviation.
(TIFF)

S1 File Data. (PDF)
