# Supplementary figures and images for: Mature Biofilm Degradation by Potential Probiotics: Aggregatibacter actinomycetemcomitans versus Lactobacillus spp
Source: PLoS One. 2016 Jul 20;11(7):e0159466. doi: 10.1371/journal.pone.0159466 (PMC4954673; doi:10.1371/journal.pone.0159466)

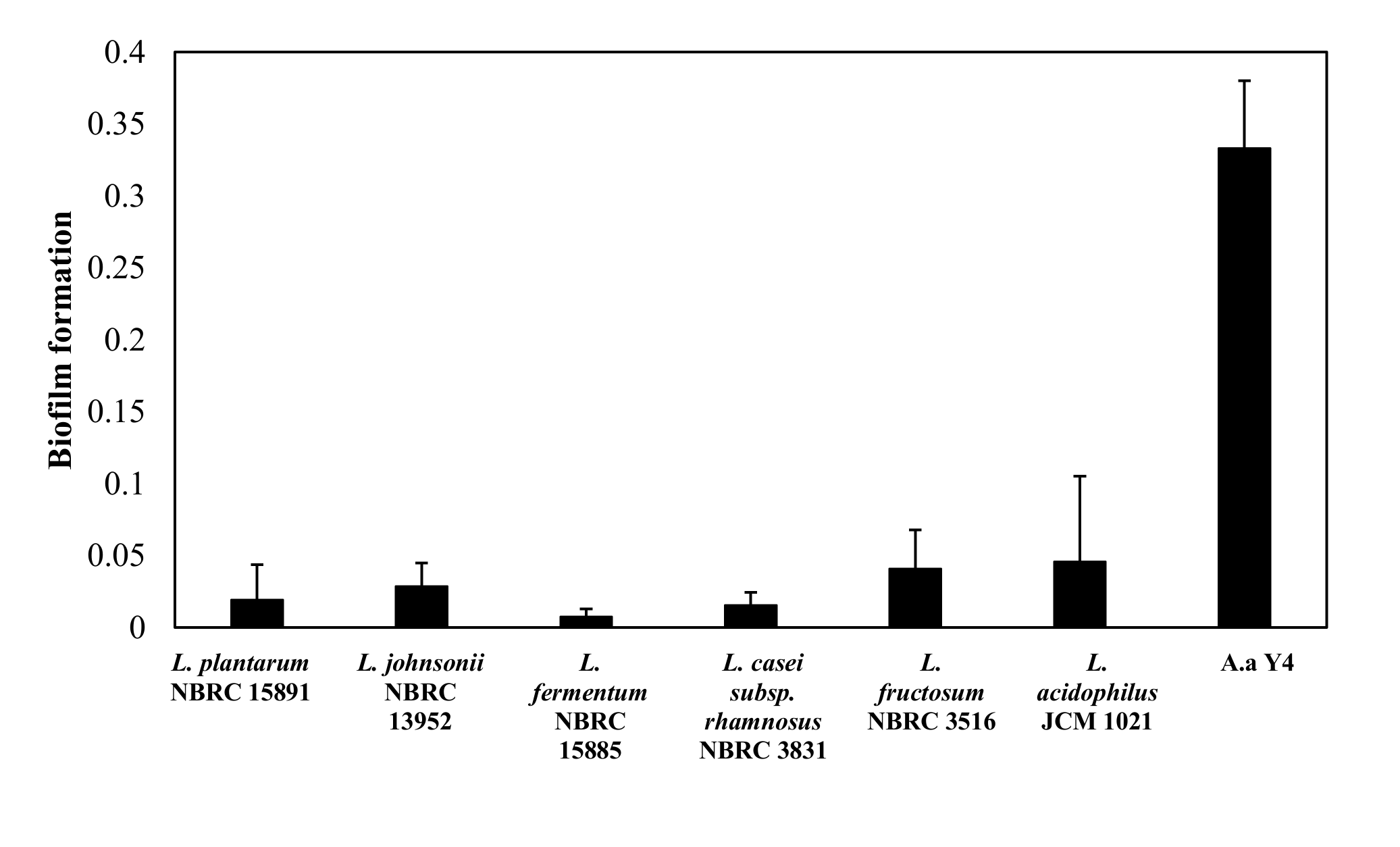

Supplement: S1 Fig — Initial OD of each sample of cell culture suspensions were 0.05 at 600nm. All samples were incubated in anaerobic condition at 37°C for 24 hour. Bars represent the mean and error bars represent standard deviation. (TIFF) [file pone.0159466.s001.tiff]

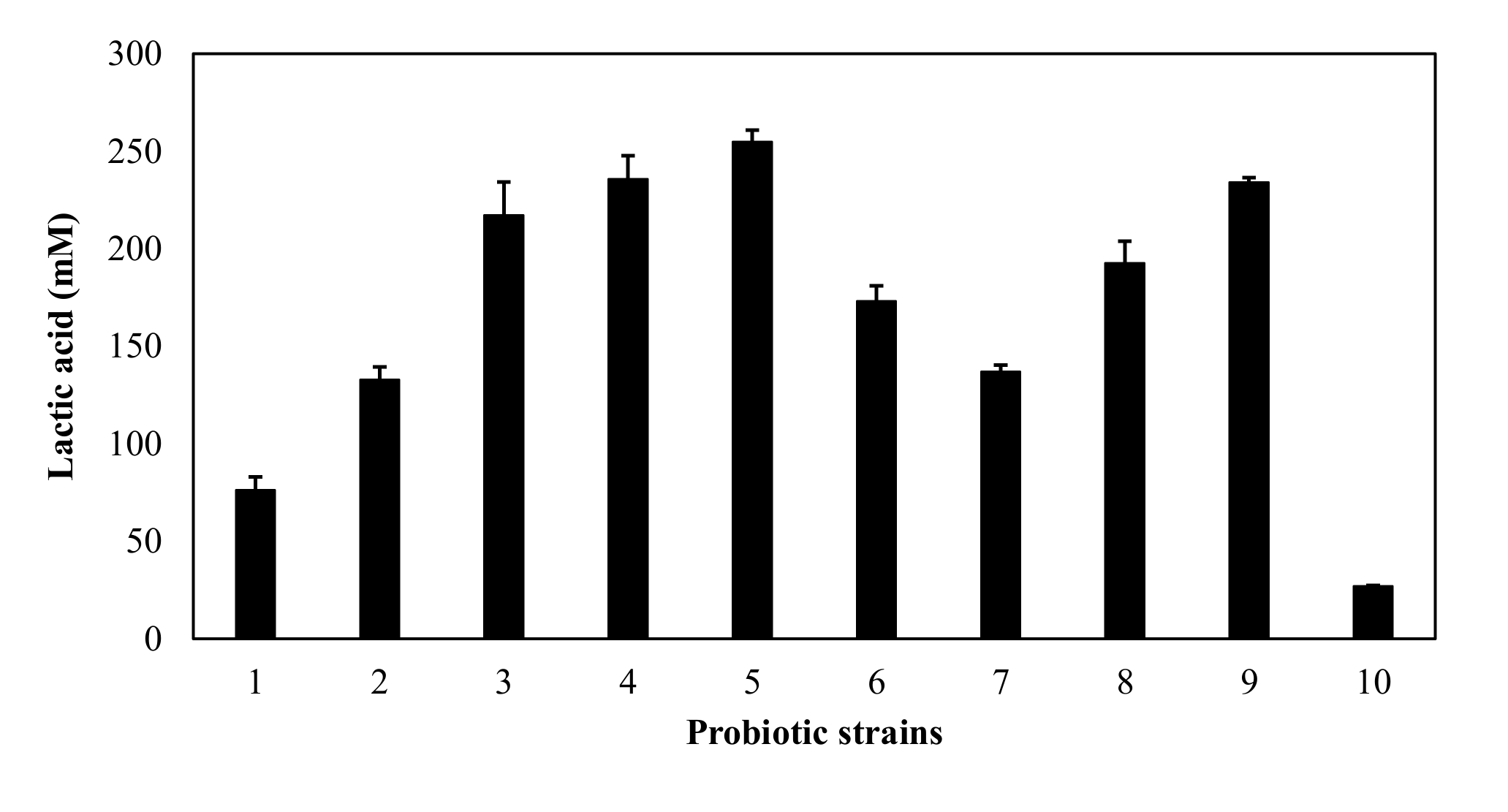

Supplement: S2 Fig — Numbers represent strains as follows; 1: Lactococcus lactic NBRC 12007, 2: L. johnsonii NBRC 13952, 3: L. casei subsp. rhamnosus NBRC 3831, 4: Lactobacillus paracasei subsp paracasei 3533, 5: Leuconostoc mesenteroides IAM 1046, 6: L. sake NBRC 3541, 7: L. fermentum NBRC 15885, 8: L. casei NBRC 15883, 9: L. plantarum NBRC 15891 and 10: Leuconostuc fructosum NBRC 3516. Bars represent the mean and error bars represent standard deviation. (TIFF) [file pone.0159466.s002.tiff]
