# Supplementary material for: Mature Biofilm Degradation by Potential Probiotics: Aggregatibacter actinomycetemcomitans versus Lactobacillus spp
Source: PLoS One. 2016 Jul 20;11(7):e0159466. doi: 10.1371/journal.pone.0159466 (PMC4954673; doi:10.1371/journal.pone.0159466)
Supplement: S1 File — (PDF) [file pone.0159466.s003.pdf]

**Biofilm formation by A.a strains with and without probiotic cell additions**						Paired T-test<br>Comparison with Control			
	**A.a Y4 (serotype b)**								
	n1	n2	n3	Ave	SD	P values	t	df	Standard error different
L. acidophilus JCM 1021	0.0472	0.0514	0.0419	0.0468	0.0047	0.0159	7.8289	2	0.0550
L. casei subsp. rhamnosus NBRC 3831	0.0306	0.0532	0.0512	0.0450	0.0125	0.0188	7.1808	2	0.0600
L. delbrueckii subsp. casei JCM 1012	0.0317	0.0388	0.0397	0.0367	0.0044	0.0174	7.4886	2	0.059
L. fermentum JCM 1137	0.0360	0.0297	0.0308	0.0322	0.0033	0.0161	7.785	2	0.0570
L. lactis NBRC 12007	0.1179	0.1153	0.1509	0.1280	0.0198	0.0369	5.0623	2	0.0690
L. fermentum NBRC 15885	0.0387	0.0347	0.0353	0.0362	0.0022	0.0150	7.6991	2	0.0570
L. casei NBRC 15883	0.0511	0.0466	0.0456	0.0478	0.0030	0.0170	7.575	2	0.0570
L. fructosum NBRC 3516	0.0409	0.0487	0.0498	0.0464	0.0049	0.0182	7.3023	2	0.0590
L. plantarum NBRC 15891	0.1839	0.2138	0.1591	0.1856	0.0274	0.0219	6.6493	2	0.0440
Leuconostoc mesenteroides IAM 1046	0.0698	0.0651	0.0591	0.0647	0.0053	0.0192	7.1121	2	0.0580
L. johnsonii NBRC 13952	0.0427	0.0467	0.0495	0.0463	0.0034	0.0274	5.9213	2	0.0690
L. sake NBRC 3541	0.0479	0.0609	0.0908	0.0665	0.0220	0.0274	5.9123	2	0.0690
L. paracasei subsp. paracasei NBRC 3533	0.0666	0.0830	0.0839	0.0778	0.0097	0.0220	6.6347	2	0.0600
A. actinomycetemcomitans Y4	0.5198	0.5461	0.3622	0.4760	0.0994				
A. naeslundii JCM 8349	0.6360	0.5880	0.5300	0.5847	0.0531	0.0970	2.9729	2	0.0370

	**A.a SUNY 75 (serotype a)**							Paired T-test-Comparison with control			
	n1	n2	n3	n4	n5	Ave	SD	P-value	t	df	Stnadard error of difference
L. fermentum JCM 1137	0.1220	0.1310	0.1300	0.1200	0.0521	0.1110	0.0333	0.0030	6.4541	4	0.0350
L.. acidophilus JCM 1021	0.1550	0.1580	0.1460	0.1510	0.1790	0.1578	0.0127	0.0076	4.9867	4	0.0360
L. fermentum NBRC 15885	0.1280	0.1120	0.0970	0.1050	0.1010	0.1086	0.0122	0.0042	5.8733	4	0.0390
L. fructosum NBRC 3516	0.0830	0.1230	0.0920	0.0910	0.1810	0.1140	0.0404	0.0092	4.7134	4	0.0047
L. plantarum NBRC 15891	0.2710	0.2510	0.2340	0.2160	0.2170	0.2378	0.0234	0.0720	2.4401	4	0.0410
L. casei subsp. rhamnosus NBRC 3831	0.4200	0.4300	0.4380	0.4430	0.4700	0.4402	0.0188	0.0347	3.1459	4	0.0330
L. johnsonii NBRC 13952	0.1314	0.1196	0.1202	0.1297	0.1318	0.1265	0.0061	0.0043	5.8445	4	0.0360
A.a SUNY 75 (serotype a)	0.2210	0.3580	0.4330	0.3470	0.3290	0.3398	0.0851				
A. naeslundii JCM 8349	0.4550	0.4480	0.3120	0.3370		0.3880	0.0741	0.5679	0.6396	3	0.0750

	**OMZ 534 (Serotype e)**		Paired-t test

	n1	n2	n3	n4	n5	Ave	SD	P-value	t	df	standard error of difference
L. fermentum JCM 1137	0.0055	0.0108	0.0135	0.0158	0.0088	0.0109	0.0040	0.0007	9.4075	4	0.0450
L.. acidophilus JCM 1021	0.1741	0.2569	0.1846	0.2261	0.2282	0.2140	0.0341	0.0104	4.5489	4	0.0490
L. fermentum NBRC 15885	0.0006	0.0019	0.0031	0.0013	0.0024	0.0019	0.0010	0.0007	9.5125	4	0.0460
L. fructosum NBRC 3516	0.0043	0.0059	0.0163	0.0127	0.0196	0.0118	0.0066	0.0006	9.6948	4	0.0440
L. plantarum NBRC 15891	0.2483	0.2424	0.1980	0.2386	0.2020	0.2259	0.0239	0.0135	4.2152	4	0.0500
L. casei subsp. rhamnosus NBRC 3831	0.3153	0.3716	0.3450	0.3539	0.2703	0.3312	0.0397	0.1400	1.8375	4	0.0570
L. johnsonii NBRC 13952	0.0180	0.0318	0.0214	0.0369	0.0216	0.0260	0.0080	0.0008	9.0436	4	0.0450
A.a OMZ534(serotype e)	0.4029	0.3009	0.4019	0.5437	0.5343	0.4367	0.1022				
A. naeslundii JCM 8349	0.7800	0.6940	0.8000	0.9160		0.7975	0.0914	0.0001	62.1513	3	0.0060

Percentage of biofilm degradation

	**A.a Y4**				
	n1	n2	n3	Ave	SD
L. acidophilus JCM 1021	90.09	89.20	91.19	90.16	1.00
L. casei subsp. rhamnosus NBRC 3831	93.56	88.82	89.24	90.54	2.63
L. delbrueckii subsp. casei JCM 1012	93.34	91.84	91.66	92.28	0.92
L. fermentum JCM 1137	92.44	93.75	93.52	93.24	0.70
L. lactis NBRC 12007	75.23	75.78	68.30	73.10	4.17
L. fermentum NBRC 15885	91.87	92.72	92.58	92.39	0.46
L. casei NBRC 15883	89.26	90.22	90.42	89.97	0.62
L. fructosum NBRC 3516	91.42	89.77	89.55	90.24	1.02
L. plantarum NBRC 15891	61.37	55.07	66.57	61.00	5.76
Leuconostoc mesenteroides IAM 1046	85.34	86.32	87.58	86.41	1.12
L. johnsonii NBRC 13952	91.03	90.19	89.60	90.28	0.72
L. sake NBRC 3541	89.94	87.21	80.93	86.02	4.62
L. paracasei subsp. paracasei NBRC 3533	86.00	82.57	82.37	83.65	2.04

	**SUNY 75**						
Percentage of degradation	n1	n2	n3	n4	n5	Average	SD
L. fermentum JCM 1137	44.80	63.41	69.98	65.42	84.15	65.55	12.66
L.. acidophilus JCM 1021	54.38	53.50	57.03	55.56	47.32	53.56	3.34
L. fermentum NBRC 15885	42.08	68.72	77.60	69.74	69.30	65.49	12.15
L. fructosum NBRC 3516	62.44	65.64	78.75	73.78	44.98	65.12	11.61

L. plantarum NBRC 15891	20.25	26.13	31.14	36.43	36.14	30.02	6.17
L. casei subsp. rhamnosus NBRC 3831	-23.60	-26.55	-28.90	-30.37	-38.32	-29.55	4.95
L. johnsonii NBRC 13952	40.53	66.59	72.25	62.64	59.94	60.39	10.76
	**OMZ 534**						
Percentage of degradation	n1	n2	n3	n4	n5	Ave	SD
L. fermentum JCM 1137	98.75	97.52	96.92	96.39	97.98	97.51	0.92
L.. acidophilus JCM 1021	60.13	41.18	57.74	48.24	47.76	51.01	7.80
L. fermentum NBRC 15885	99.86	99.57	99.29	99.71	99.44	99.58	0.22
L. fructosum NBRC 3516	99.01	98.64	96.28	97.09	95.52	97.31	1.50
L. plantarum NBRC 15891	43.16	44.49	54.66	45.36	53.75	48.28	5.47
L. casei subsp. rhamnosus NBRC 3831	27.80	14.92	21.01	18.98	38.11	24.16	9.08
L. johnsonii NBRC 13952	95.87	92.71	95.09	91.55	95.05	94.06	1.84

Comparisons of cell addition and supernatant addition on Y4 biofilm

**Biofilm values Y4**	Cells addition					vs	Supernatant addition				
	n1	n2	n3	Ave	SD		n1	n2	n3	Ave	SD
L. johnsonii NBRC 13952	0.0427	0.0467	0.0495	0.0463	0.0034		0.1940	0.1768	0.1648	0.1785	0.0147
L. acidophilus JCM 1021	0.0472	0.0514	0.0419	0.0468	0.0047		0.3024	0.2868	0.2566	0.2820	0.0233
L. fructosum NBRC 3516	0.0409	0.0487	0.0498	0.0464	0.0049		0.1642	0.1535	0.1608	0.1595	0.0055
L. casei subsp. rhamnosus NBRC 3831	0.0306	0.0532	0.0512	0.0450	0.0125		0.1690	0.1393	0.1584	0.1555	0.0150
L. fermentum NBRC 15885	0.0387	0.0347	0.0353	0.0362	0.0022		0.1642	0.1535	0.1608	0.1595	0.0055
A. actinomycetemcomitans Y4 (control)	0.5198	0.5461	0.3622	0.4760	0.0994						

Percentage of Degradation	Cell addition					Vs	Supernatant addition				
	n1	n2	n3	Ave	SD		n1	n2	n3	Ave	SD
L. johnsonii NBRC 13952	91.03	90.19	89.60	90.28	0.72		59.24	62.86	65.38	62.49	3.09
L. acidophilus JCM 1021	90.09	89.20	91.19	90.16	1.00		36.46	39.74	46.08	40.76	4.89
L. fructosum NBRC 3516	91.42	89.77	89.55	90.24	1.02		65.51	67.75	66.22	66.49	1.15
L. casei subsp. rhamnosus NBRC3831	93.56	88.82	89.24	90.54	2.63		64.50	70.74	66.73	67.32	3.16
L. fermentum NBRC 15885	91.87	92.72	92.58	92.39	0.46		65.51	67.75	66.22	66.49	1.15

Comparison of nutrient rich medium and co-aggregation buffer condition

	**Nutrient rich Medium**					**Percentage of Degradation**					**Co-aggregation buffer condition**						**Percentage of Degradation**					
	n1	n2	n3	Ave	SD	n1	n2	n3	Ave	SD	n1	n2	n3	n4	Ave	SD	n1	n2	n3	n4	Ave	SD
L. johnsonii NBRC 13952	0.0427	0.0467	0.0495	0.0463	0.0034	91.03	90.19	89.60	90.28	0.72	0.0636	0.0664	0.0733	0.0744	0.0694	0.0052	94.09	93.83	93.19	93.08	93.55	0.49
L. acidophilus JCM 1021	0.0472	0.0514	0.0419	0.0468	0.0047	90.09	89.20	91.19	90.16	1.00	0.1431	0.1438	0.1390	0.1265	0.1381	0.0080	86.70	86.63	87.08	88.24	87.16	0.74
L. fructosum NBRC 3516	0.0409	0.0487	0.0498	0.0464	0.0049	91.42	89.77	89.55	90.24	1.02	0.1564	0.2734	0.1379	0.1687	0.1841	0.0609	85.46	74.59	87.18	84.32	82.89	5.66
L. casei subsp. rhamnosus NBRC 3831	0.0306	0.0532	0.0512	0.0450	0.0125	93.57	88.82	89.24	90.54	2.63	0.0794	0.0931	0.0832	0.0847	0.0851	0.0058	92.63	91.34	92.27	92.13	92.09	0.54
L. fermentum NBRC 15885	0.0387	0.0347	0.0353	0.0362	0.0022	91.87	92.72	92.58	92.39	0.46	0.0402	0.0599	0.0504	0.0391	0.0474	0.0098	96.26	94.43	95.32	96.36	95.59	0.91
L. plantarum NBRC 15891	0.1839	0.2138	0.1591	0.1856	0.0274	61.37	55.08	66.57	61.01	5.75	0.0890	0.0799	0.0867	0.0902	0.0864	0.0046	91.73	92.58	91.94	91.62	91.97	0.43
A. actinomycetemcomitans Y4 (control)	0.5198	0.5461	0.3622	0.4760	0.0994						1.0932	0.9817	0.9717	0.9817	1.0071	0.0576						

Autoclaved cells	n1	n2	n3	n4	n5	n1	n2	n3	n4	n5	
L. johnsonii NBRC 13952	0.278	0.218	0.199	0.157	0.266	-110.28744	-64.901664	-50.529501	-18.759455	-101.2103	-92.1331
L. acidophilus JCM 1021	0.106	0.215	0.28	0.148	0.211	-113.31316	-125.41604	-100.45386	-18.759455	-112.7458	-117.158
L. fructosum NBRC 3516	0.115	0.139	0.123	0.147	0.16	-24.054463	-42.208775	-40.695915	-31.618759	-28.59304	-31.6188
L. casei subsp. rhamnosus NBRC 3831	0.108	0.162	0.121	0.137	0.121	18.305598	-22.541604	8.4720121	-3.6308623	8.472012	1.412002
L. fermentum NBRC 15885	0.064	0.066	0.055	0.061	0.078	51.588502	50.075643	58.396369	53.857791	40.99849	47.55421
A. actinomycetemcomitans Y4	0.114	0.103	0.117	0.116	0.085						

For CFU, Samples were collected from the supernatant of a biofilm

	CFU/ml			Log CFU/ml					Biofilm value					
Bacteria	n1	n2	n3	n1	n2	n3	Ave	SD	n1	n2	n3	n4	Ave	SD
A.a biofilm control	480	800	1000	2.6812412	2.90309	3	2.861444	0.163409	1.093	0.982	0.972	0.982	1.007058	0.057598
A.a + L. fermentum NBRC 15885	492000	602000	6000000	5.6919651	5.7795965	6.77815125	6.083238	0.603406	0.040	0.060	0.050	0.039	0.04742	0.009758
A.a + L. casei subsp. rhamnosus 3831	28000	100000	60000	4.447158	5	4.77815125	4.74177	0.278211	0.079	0.093	0.083	0.085	0.085089	0.005812
A.a+ L. fructosum NBRC 3516	18000	62000	25000	4.2552725	4.7923917	4.39794001	4.481868	0.278221	0.156	0.273	0.138	0.169	0.18412	0.060851
A.a+ L. acidophilus JCM 1021	6000	60000	60000	3.7781513	4.7781513	4.77815125	4.444818	0.57735	0.143	0.144	0.139	0.127	0.138119	0.008012
A.a + L. johnsonii NBRC 13952	260000000	68000000	60000000	8.4149733	7.8325089	7.77815125	8.008545	0.353025	0.064	0.066	0.073	0.074	0.069433	0.005236

Comparison of adjusted and non-adjusted pH of probiotic supernatant on biofilm degradation

Biofilm values					Biofilm values		Percentage of Degradation					Paired T-test			
n1	n2	n3	Bacteria	Condition	Ave	SD	n1	n2	n3	Average	SD	P value	t	df	standard error of difference
0.5198	0.5461	0.3622	A. actinomycetemcomitans Y4 control		0.4760	0.0994									
0.1940	0.1768	0.1648	L. johnsonii NBRC 13952	untreated	0.1785	0.0147	59.24	62.86	65.38	62.49	3.09				

0.1759	0.1627	0.1369		pH 6.5	0.1585	0.0198	63.05	65.82	71.23	66.70	4.16	0.04	4.65	2.00	0.88
0.3024	0.2868	0.2566	L. acidophilus JCM 1021	untreated	0.2820	0.0233	36.46	39.74	46.08	40.76	4.89				
0.2021	0.2286	0.3649		pH 6.5	0.2652	0.0873	57.55	51.98	23.35	54.76	3.94	0.82	0.26	2	13.38
0.1642	0.1535	0.1608	L. fructosum NBRC 3516	untreated	0.1595	0.0055	65.51	67.75	66.22	66.49	1.15				
0.1495	0.1286	0.1498		pH 6.5	0.1426	0.0121	68.59	72.98	68.53	70.03	2.55	0.06	4.05	2	0.87
0.1398	0.1606	0.2297	L. casei subsp. rhamnosus NBRC 3831	untreated	0.1767	0.0471	70.63	66.27	51.75	68.45	3.08				
0.2087	0.1993	0.3055		pH 6.5	0.2378	0.0588	56.16	58.14	35.83	57.15	1.40	0.03	5.37	2	2.39
0.1690	0.1393	0.1584	L. fermentum NBRC 15885	untreated	0.1555	0.0150	64.50	70.74	66.73	67.32	3.16				
0.2115	0.2520	0.1982		pH 6.5	0.2206	0.0280	55.56	47.07	58.37	53.67	5.89	0.11	2.73	2	5.01

Biofilm degradation of by lactic acid influence

**Biofilm values**				**Biofilm values**	
n1	n2	n3		Average	SD
0.3270	0.3217	0.2804	250 mM (lactic acid)	0.30971	0.025533
0.3656	0.3140	0.3733	200 mM (lactic acid)	0.35098	0.032242
0.3173	0.2987	0.3951	150 mM (lactic acid)	0.33700	0.051133
0.3131	0.3805	0.3605	100 mM (lactic acid)	0.34680	0.04761

Comparison between lactic acid addition with cells addition			
T-test			
P value	t	df	standard error of difference
0.0001	24.3252	7	0.011

Biofilm degradation by probiotics bacteria

Probiotic strains	n1	n2	n3	n4	n5	n6	Ave	SD
L. sake NBRC 3541	0.0479	0.0609	0.0908	0.0848	0.0797	0.0911	0.0759	0.017868
L. fermentum NBRC 15885	0.0387	0.0347	0.0353	0.0342	0.0393	0.0511	0.0389	0.002384
L. acidophilus JCM 1021	0.0472	0.0514	0.0419				0.0468	0.004742
L. casei NBRC 15883	0.0511	0.0466	0.0456	0.0863	0.0751	0.0832	0.0647	0.018607
L. johnsonii NBRC 13952	0.0427	0.0467	0.0495	0.0699	0.0664	0.1423	0.0696	0.012297

Effect of enzymes on biofilm degradation (in co-aggregation buffer)

**Enzyme degradation activity**							% Degradation
	n1	**n2**	**n3**	**n4**	**Ave**	**SD**	**%**
A.actinomycetemcomitans Y4 control (serotype b)	1.0932	0.9807	0.9717	0.9817	1.0068	0.0577	
Proteinase K	0.5147	0.7068	0.5000	0.6336	0.5888	0.0733	41.52114
Lipase	0.1346	0.0943	0.1018	0.0903	0.0955	0.0058	90.51587
Amylase	0.4776	0.4662	0.4565	1.4865	0.4668	0.0106	53.63862
combine	0.0541	0.0615	0.1118	0.0775	0.0762	0.0257	92.43113
A. actinomycetemcomitans SUNY 75 (serotype a)	1.7195	1.6988	1.6543	1.3474	1.6050	0.1739	
Proteinase K	0.7869	0.7429	0.7231	0.7581	0.7527	0.0269	53.1015
Lipase	1.1765	1.0390	1.0267	1.0000	1.0605	0.0790	33.9237
Amylase	1.3684	1.2530	1.2500	1.3659	1.3093	0.0668	18.42232
combine	0.9219	0.7059	0.7761	0.7361	0.7850	0.0957	51.09053
A. actinomycetemcomitans OMZ534 (serotype e)	0.6052	0.4060	0.4007	0.4382	0.4625	0.0965	
Proteinase K	0.1579	0.1786	0.1579	0.1429	0.1593	0.0147	65.55578
Lipase	0.1905	0.1051	0.1252	0.1654	0.1465	0.0386	68.31675
Amylase	0.2708	0.1962	0.2345	0.1867	0.2220	0.0385	51.98998
combine	0.0702	0.1379	0.0833	0.0172	0.0772	0.0496	83.31453

**Lipase Activity Profile from supernatant**	Absorbance			BALB			IU/L				
	n1	n2	n3	n1	n2	n3	n1	n2	n3	Ave	SD
Positive control	0.030	0.028	0.031	30.000	28.000	31.000	4.410	4.116	4.557	4.361	0.225
L. fermentum NBRC 15885	0.037	0.036	0.031	37.000	36.000	31.000	5.439	5.292	4.557	5.096	0.473
L. casei subsp. rhamnosus NBRC 3831	0.069	0.064	0.065	69.000	64.000	65.000	10.143	9.408	9.555	9.702	0.389
L. fructosum NBRC 3516	0.037	0.030	0.034	37.000	30.000	34.000	5.439	4.410	4.998	4.949	0.516
L. acidophilus JCM 1021	0.042	0.064	0.080	42.000	64.000	80.000	6.174	9.408	11.760	10.584	1.663
L. johnsonii NBRC 13952	0.069	0.068	0.081	69.000	68.000	81.000	10.143	9.996	11.907	10.682	1.063
A. naeslundii JCM 8349	0.057	0.064	0.054	56.667	64.333	54.000	8.33	9.457	7.938	8.575	0.789
negative control	0.008	0.005	0.009	8.000	5.000	9.000	1.176	0.735	1.323	1.078	0.306
*0.001 Abs=1BALB<br>IU/L=BALBx0.147 (from kit manual)											

Influence of Lipase inhibitor on biofilm degradation activity											
	A. actinomycetemcomitans Y4 (serotype b)							Paired T-test with and without Lipase inhibitor			
	n1	n2	n3	n4	n5	Ave	SD	P value	t	df	standard error of difference
L. fermentum NBRC 15885 supernatnat +Li	0.085	0.079	0.058	0.055	0.118	0.079	0.025	0.0105	9.6916	2	0.009
L. fermentum NBRC 15885 supernatant	0.164	0.154	0.161			0.159	0.005				
L. casei subsp. rhamnosus NBRC 3831 supernatnat +Li	0.864	1.519	1.497	1.594	1.926	1.480	0.385	0.0359	5.1333	2	0.222
L. casei subsp. rhamnosus NBRC 3831 supernatant	0.169	0.139	0.158			0.156	0.015				
L. fructosum NBRC 3516 supernatnat +Li	0.057	0.113	0.098	0.092	0.040	0.080	0.031	0.0716	3.5347	2	0.02
L. fructosum NBRC 3516 supernatnat	0.164	0.154	0.161			0.159	0.005				
L. acidophilus JCM 1021 supernatnat +Li	1.565	1.721	2.032	1.992	2.041	1.870	0.215	0.001	9.9052	2	0.15
L. acidophilus JCM 1021 supernatnat	0.302	0.287	0.257			0.282	0.023				
L. johnsonii NBRC 13952 supernatnat +Li	0.426	0.391	0.551	0.563		0.483	0.087	0.0366	5.0811	2	0.055
L. johnsonii NBRC 13952 supernatnat	0.194	0.177	0.165			0.179	0.015				
L. plantarum NBRC 15891 supernatnat +Li	2.293	2.088	2.000	2.053		2.109	0.128	0.0018	23.7927	2	0.089
L. plantarum NBRC 15891 supernatnat	0.018	0.015	0.026			0.020	0.006				
A. naeslundii JCM 8349 supernatant +Li	0.667	0.909	0.520	0.382		0.619	0.225	0.3105	1.2172	3	0.02
A. naeslundii JCM 8349 supernatant	0.581	0.676	0.615	0.550		0.605	0.005				
A.a control	0.520	0.546	0.362	0.457	0.376	0.452	0.083				

Influence of Lipase inhibitor on biofilm degradation activity											
	**A. actinomycetemcomitans SUNY 75 (serotype a)**							Paired t-test comparison with and without lipase inhibitor			
	n1	n2	n3	n4	n5	Ave	SD	P value	t	df	standard error of difference
L. fermentum NBRC 15885 supernatnat +Li	0.1200	0.1190	0.1130	0.1290	0.1110	0.1184	0.0071	0.9284	0.0976	3	0.003
L. fermentum NBRC 15885 supernatant	0.1150	0.1170	0.1200	0.1280		0.1200	0.0057				
L. casei subsp. rhamnosus NBRC 3831 supernatnat +Li	0.3600	0.3260	0.3720	0.3670		0.3563	0.0208	0.01	9.9348	2	0.012
L. casei subsp. rhamnosus NBRC 3831 supernatant	0.2360	0.2320	0.2410			0.2363	0.0045				
L. fructosum NBRC 3516 supernatant +Li	0.1028	0.1087	0.1055	0.0967		0.1034	0.0051	0.3537	1.0946	3	0.009
L. fructosum NBRC 3516 supernatnat	0.1221	0.0960	0.1098	0.1256		0.1134	0.0134				
L. acidophilus JCM 1021 supernatnat +Li	0.2520	0.2410	0.2630	0.2750		0.2578	0.0146	0.775	0.3127	3	0.05
L. acidophilus JCM 1021 supernatnat	0.3690	0.3240	0.2070	0.1930		0.2733	0.0867				
L. johnsonii NBRC 13952 supernatnat +Li	0.2140	0.2080	0.1920	0.2240		0.2095	0.0134	0.0104	5.7624	3	0.006
L. johnsonii NBRC 13952 supernatnat	0.1780	0.1774	0.1700	0.1732		0.1747	0.0038				
L. plantarum NBRC 15891 supernatnat +Li	0.4180	0.3760	0.3720	0.3720		0.3845	0.0224	0.3095	1.2204	3	0.027
L. plantarum NBRC 15891 supernatnat	0.3750	0.3673	0.2680	0.3950		0.3513	0.0568				
A. naeslundii JCM 8349 supernatant +Li	0.3890	0.2980	0.3460	0.2970		0.3325	0.0441	0.7537	0.3437	3	0.023
A. naeslundii JCM 8349 supernatant	0.3400	0.3230	0.2990	0.3360		0.3245	0.0185				
A.actinomycetemcomitans SUNY 75 (control)	0.2210	0.2210	0.2810	0.2040		0.2318	0.0338				

Influence of Lipase inhibitor on biofilm degradation activity											
	**A. actinomycetemcomitans OMZ (serotype e)**							Paired t-test			
	n1	n2	n3	n4	n5	Ave	SD	P value	t	df	standard error of difference
L. fermentum NBRC 15885 supernatnat +Li	0.0487	0.0299	0.0549	0.0363		0.042428	0.011415	0.387	1.0097	3	0.008
L. fermentum NBRC 15885 supernatant	0.0270	0.0339	0.0314	0.0441		0.0341	0.00725				
L. casei subsp. rhamnosus NBRC 3831 supernatnat +Li	1.1317	0.9414	0.7417	1.4672		1.070488	0.308674	0.335	1.1457	3	0.219
L. casei subsp. rhamnosus NBRC 3831 supernatant	0.6420	0.6609	1.3655	0.5203		0.797205	0.38395				
L. fructosum NBRC 3516 supernatnat +Li	0.0386	0.0379	0.0265	0.0203		0.030811	0.008915	0.3872	1.0091	3	0.007
L. fructosum NBRC 3516 supernatnat	0.0360	0.0660	0.0229	0.0281		0.038234	0.019271				
L. acidophilus JCM 1021 supernatnat +Li	2.9771	2.8061	3.0112	2.5196		2.828512	0.224633	0.0018	10.6443	3	0.164
L. acidophilus JCM 1021 supernatnat	0.8442	0.9183	1.5471	1.0415		1.087777	0.316837				
L. johnsonii NBRC 13952 supernatnat +Li	2.7150	3.1988	3.1389	4.1079		3.290158	0.586161	0.002	10.226	3	0.309
L. johnsonii NBRC 13952 supernatnat	0.1294	0.1415	0.1727	0.0710		0.128641	0.042517				
L. plantarum NBRC 15891 supernatnat +Li	0.3756	0.9327	0.3583	0.7378		0.601102	0.281937	0.4635	0.8381	3	0.139
L. plantarum NBRC 15891 supernatnat	0.4560	0.4448	0.4691	0.5693		0.484823	0.057219				
A. naeslundii JCM 8349 supernatant +Li	0.2550	0.3390	0.1780	0.2160		0.247	0.06892	0.8376	0.2234	3	0.04
A. naeslundii JCM 8349 supernatant	0.2490	0.2170	0.2420	0.2440		0.238	0.014306				
A.actinomycetemcomitans OMZ534 control	0.4029	0.3009	0.4019	0.5437	0.534286	0.436745	0.102168				

Lactic acid concentration of culture supernatant of biofilm assay after probiotic strains addition

	Lactic acid										
Day 4 biofilm supernatant	Area {y}	x	[Lactic acid] mM	Area {y}	x	[Lactic acid] mM	Area {y}	x	[Lactic acid] mM	Ave	SD
L. lactis 12007	260721	14.1712	70.8558	272854	14.83063376	74.15316882	308541	16.77036	83.85177737	76.2869	6.755628
L. jonhsonii 13952	*461351	25.0761	125.3807	501489	27.25779976	136.2889988	504782	27.43679	137.183933	132.9512	6.571479
L. casei 3831	731318	39.7499	198.7493	854121	46.42466572	232.1233286	812795	44.17844	220.8922165	217.2550	16.98171
L. paracasei subsp. paracasei 3533	819712	44.5544	222.7720	874552	47.53516687	237.6758343	907542	49.3283	246.6414828	235.6965	12.0572
Leuconostoc mesenteroides IAM 1046	912357	49.5900	247.9500	953325	51.81677356	259.0838678	947892	51.52147	257.6073486	254.8804	6.047113
L. sake 3541	642897	34.9439	174.7193	662871	36.02951408	180.1475704	605278	32.89912	164.4955973	173.1208	7.947475
L. fermentum 15885	489588	26.6109	133.0547	509742	27.70638113	138.5319056	512785	27.87178	139.3588977	136.9818	3.426054
L. casei 15883	671656	36.5070	182.5351	700248	38.0610936	190.305468	754125	40.98951	204.9475486	192.5960	11.38046
L. plantarum 15891	857849	46.6273	233.1365	871547	47.37183389	236.8591695	854172	46.42744	232.1371888	234.0443	2.488443
Leuconostoc fructosum 3516	99303	5.3975	26.9874	100542	5.464833134	27.32416567	99311	5.397924	26.98961844	27.1004	0.193782

Biofilm formation by probiotic strains in mono-culture compared to A.a Y4 in monoculture

	n1	n2	n3	n4	n5	n6	Average	SD
L. plantarum NBRC 15891	0.0031	0.0045	0.0022	0.0310	0.0112	0.0640	0.0193	0.0244
L. johnsonii NBRC 13952	0.0112	0.0128	0.0541	0.0302	0.0389	0.0248	0.0287	0.0163
L. fermentum NBRC 15885	0.0175	0.0099	0.0029	0.0035	0.0056	0.0058	0.0075	0.0055
L. casei subsp. rhamnosus 3831	0.0258	0.0048	0.0192	0.0040	0.0202	0.0189	0.0155	0.0089
L. fructosum NBRC 3516	0.0939	0.0377	0.0233	0.0197	0.0366	0.0344	0.0409	0.0270
L. acidophilus JCM 1021	0.0121	0.0193	0.1592	0.0117	0.0652	0.0070	0.0457	0.0596
A.a only	0.3990	0.3331	0.3524	0.2749	0.3063		0.3331	0.0470
